# Supplementary figures and images for: Sea lamprey nests promote the diversity of benthic macroinvertebrate assemblages
Source: PLoS One. 2022 Dec 15;17(12):e0274719. doi: 10.1371/journal.pone.0274719 (PMC9754182; doi:10.1371/journal.pone.0274719)

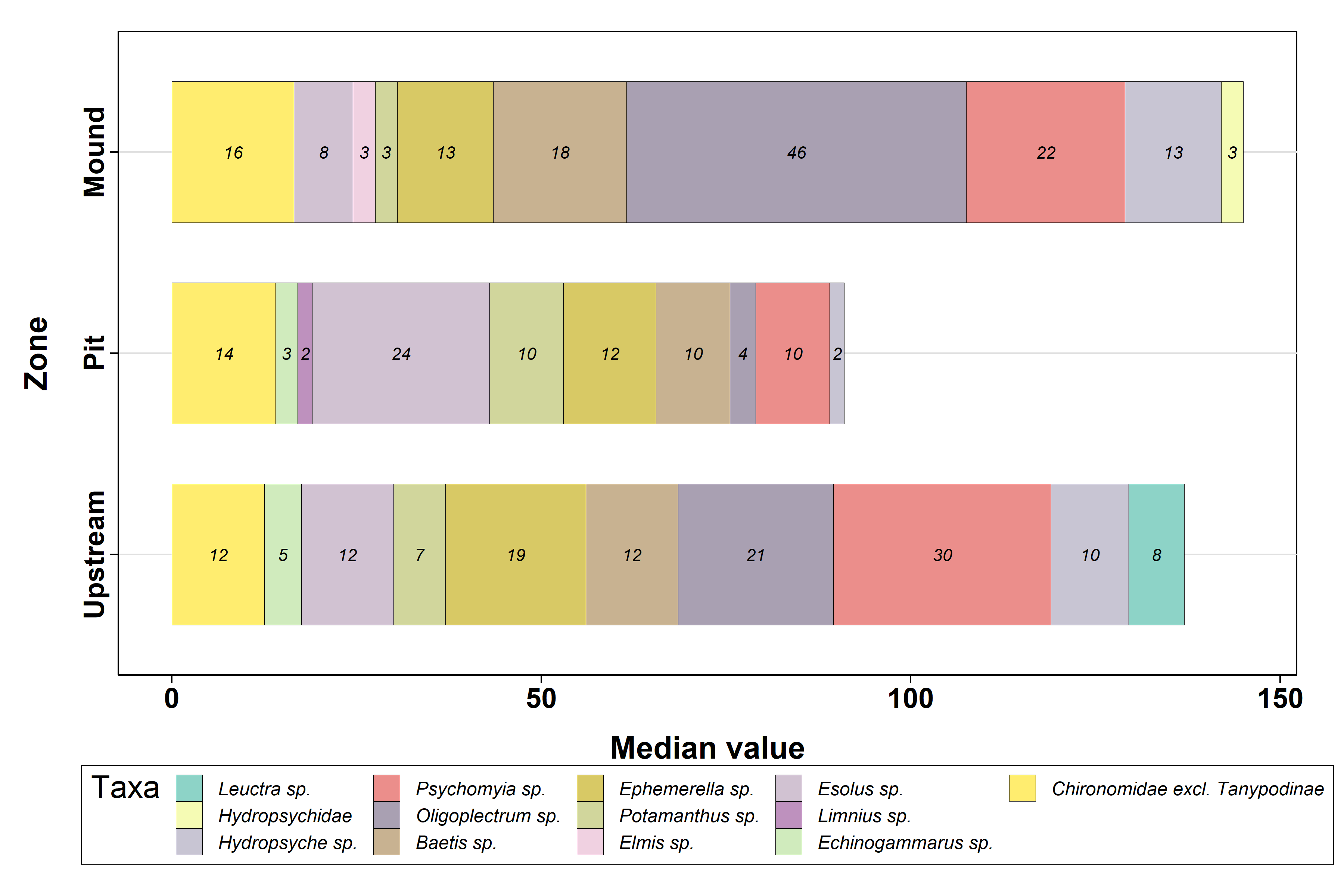

Supplement: S1 Fig — (TIFF) [file pone.0274719.s001.tiff]
