# Supplementary material for: Sea lamprey nests promote the diversity of benthic macroinvertebrate assemblages
Source: PLoS One. 2022 Dec 15;17(12):e0274719. doi: 10.1371/journal.pone.0274719 (PMC9754182; doi:10.1371/journal.pone.0274719)
Supplement: S1 Table — With, for column names: 1 Leuctra; 2 Perlodes; 3 Perla; 4 Rhyacophila; 5 Glossosomatidae; 6 Agapetus; 7 Hydroptila; 8 Hydropsychidae; 9 Hydropsyche; 10 Polycentropodidae; 11 Polycentropus; 12 Psychomyiidae; 13 Psychomyia; 14 Oligoplectrum; 15 Micrasema; 16 Goeridae; 17 Lepidostoma; 18 Athripsodes; 19 Ceraclea; 20 Sericostomatidae; 21 Baetis; 22 Oligoneuriella; 23 Heptageniidae; 24 Epeorus; 25 Rhithrogena; 26 Ecdyonurus; 27 Ephemerella ignita; 28 Caenis; 29 Ephemera; 30 Potamanthus; 31 Hydraena; 32 Stenelmis; 33 Elmis; 34 Esolus; 35 Oulimnius; 36 Limnius; 37 Normandia; 38 Micronecta; 39 Aphelocheirus; 40 Blephariceridae; 41 Limoniidae; 42 Simuliidae; 43 Tanypodinae; 44 Ceratopogonidae; 45 Empididae; 46 Athericidae; 47 Gammaridae; 48 Echinogammarus; 49 Hydracarina; 50 Piscicolidae; 51 Erpobdellidae; 52 Oligochaeta; 53 Theodoxus; 54 Potamopyrgus; 55 Radix; 56 Planorbidae; 57 Ancylus; 58 Dugesiidae; 59 Nematoda; 60 Hydroporinae; 61 Hydrophilinae; 62 Prostoma; 63 Hydrozoa; 64 Ostracoda; 65 Copepoda; 66 Agapetinae; 67 Physella; 68 Leuctra geniculata; 69 Chironomidae excl. Tanypodinae. (PDF) [file pone.0274719.s003.pdf]

S 1. List of taxa found in each zone of each nest. With, for column names: *1 Leuctra; 2 Perlodes; 3 Perla; 4 Rhyacophila; 5 Glossosomatidae; 6 Agapetus; 7 Hydroptila; 8 Hydropsychidae; 9 Hydropsyche; 10 Polycentropodidae; 11 Polycentropus; 12 Psychomyiidae; 13 Psychomyia; 14 Oligoplectrum; 15 Micrasema; 16 Goeridae; 17 Lepidostoma; 18 Athripsodes; 19 Ceraclea; 20 Sericostomatidae; 21 Baetis; 22 Oligoneuriella; 23 Heptageniidae; 24 Epeorus; 25 Rhithrogena; 26 Ecdyonurus; 27 Ephemerella ignita; 28 Caenis; 29 Ephemera; 30 Potamanthus; 31 Hydraena; 32 Stenelmis; 33 Elmis; 34 Esolus; 35 Oulimnius; 36 Limnius; 37 Normandia; 38 Micronecta; 39 Aphelocheirus; 40 Blephariceridae; 41 Limoniidae; 42 Simuliidae; 43 Tanypodinae; 44 Ceratopogonidae; 45 Empididae; 46 Athericidae; 47 Gammaridae; 48 Echinogammarus; 49 Hydracarina; 50 Piscicolidae; 51 Erpobdellidae; 52 Oligochaeta; 53 Theodoxus; 54 Potamopyrgus; 55 Radix; 56 Planorbidae; 57 Ancylus; 58 Dugesiidae; 59 Nematoda; 60 Hydroporinae; 61 Hydrophilinae; 62 Prostoma; 63 Hydrozoa; 64 Ostracoda; 65 Copepoda; 66 Agapetinae; 67 Physella; 68 Leuctra geniculata; 69 Chironomidae excl. Tanypodinae.*

Nest	Zone	Num. of ind.	Num. of taxa	1	2	3	4	5	6	7	8	9	10	11	12	13	14	15	16	17	18	19	20	21
**1**	Mound	215	21	10							3	9	2			24	9							11
**1**	Pit	157	15	12							3					5	37							3
**1**	Upstream	169	21	18			1	2			1	9				33	2							3
**2**	Mound	272	25	7							14	17			1	41	48							22
**2**	Pit	133	18	1			1		1		1	1				9	1							12
**2**	Upstream	297	26	3			4				19	14	1			30	96							36
**3**	Mound	184	18				3				9	17				15	68							11
**3**	Pit	73	15									1				9	8							11
**3**	Upstream	291	24	9					1			19		2		96	6							25
**4**	Mound	101	15								1	3				24	1							20
**4**	Pit	144	23	9			2				6	3				26								20
**4**	Upstream	182	23	4							3	16	1			50	4			1				24
**5**	Mound	142	19	3			2					3		1		36	5							26
**5**	Pit	92	17	1							1	1				7	2							11
**5**	Upstream	202	24	6			1		2		7	19				52	11							23
**6**	Mound	146	21				1		1		2	9				22	19				1			17
**6**	Pit	83	15	5												15								2
**6**	Upstream	124	21	7	1							15				6	1							8
**7**	Mound	198	20	2			1				7	13				25	92							13
**7**	Pit	166	18	7					1			6				17	46							28
**7**	Upstream	58	20	4								2			1	13	3							6
**8**	Mound	122	19	1					1		3	7				16	35							8
**8**	Pit	123	18	1							5	3				8	12							21
**8**	Upstream	107	11	2			1					11				13	48							11
**9**	Mound	163	19	3			1				10	13				19	48							13
**9**	Pit	81	15								1	1				7	7							10
**9**	Upstream	189	23	9			1		1		5	10		3	1	33	24							17
**10**	Mound	178	23	6					15		4	8		1		20	19							11
**10**	Pit	243	21	2							1	2				19	1				1			12
**10**	Upstream	202	27	10					3	1	3	8		1		52	3	1		1				17
**11**	Mound	209	26	23				1	3		1	3		1		23	3							13
**11**	Pit	234	18	6			1					1				11	5							9
**11**	Upstream	217	20	17					2		2	6				32	3				1			11
**12**	Mound	217	20	9			4				6	17				48	74							7
**12**	Pit	146	17				1					3				6	2							6
**12**	Upstream	196	22	14		1		1			1	14				29	25							6
**13**	Mound	162	17	1			1				3	8				15	46							9
**13**	Pit	323	24	2								5				11	1			1	1			10
**13**	Upstream	274	28	10							3	7				28	11			1			1	9
**14**	Mound	213	26	8	1		3				5	9		2		27	50							24
**14**	Pit	99	18	1					1		1					10	5							10
**14**	Upstream	144	21	2			2				1	12		1		50	5							14
**15**	Mound	174	23	9					1			17				19	4			1	1			21

Nest	Zone	22	23	24	25	26	27	27	29	30	31	32	33	34	35	36	37	38	39	40	41	42	43	44	45
**1**	Mound		3			6	28			22			2	14		2									
**1**	Pit					2	3			25			1	25		11									
**1**	Upstream					2	14			35			2	12		2									1
**2**	Mound		3	1		2	52			5	1		9	12		2				1	1	1			
**2**	Pit						12			5			1	52		4									
**2**	Upstream	1	5	1		2	23			6	2		12	24		3				2	1				
**3**	Mound					3	13			3			2	9		1					1				
**3**	Pit					1	11			11				7		1						1			
**3**	Upstream		2			2	15			20			4	27		2				1	2				
**4**	Mound					1	13			8			3	7					1		1				
**4**	Pit					4	13			10			23	6		4					1				
**4**	Upstream		5			5	29			1			2	7		1					2	1			
**5**	Mound		1			1	19			6			1	16								1			1
**5**	Pit				1	2	8			8			1	40		2				1					
**5**	Upstream		1			5	19			4			4	11		1				1	1	1			
**6**	Mound		2			5	25			5			1	8		1									
**6**	Pit		1				2			19				17		1				1					
**6**	Upstream					1	19			19			5	10	1	8					1				2
**7**	Mound						11			2			5	6		1			1		1	1			
**7**	Pit					3	13			5				8		4				1					
**7**	Upstream		1				5							6		1				1	1	1			1
**8**	Mound		2				8			9			3	5		2				1					
**8**	Pit		1			3	14			13				13		2									
**8**	Upstream						3							2											
**9**	Mound		1			3	20			1			4	6	1	1					1				
**9**	Pit						10			8		1	2	28	2										
**9**	Upstream		2			3	31			2			5	15		2					4				
**10**	Mound		1			6	11			12			4	11								2			
**10**	Pit						23			76			1	46		10					1				2
**10**	Upstream		1			1	8			16			1	22	1	6									
**11**	Mound		1			3	11			32	1		7	19		5					1				1
**11**	Pit						26	1		92			5	27		3									
**11**	Upstream					3	20			46			6	12		7					3				
**12**	Mound					1	6						7	8		2					2	2			1
**12**	Pit						23			36				48		2		1					1		
**12**	Upstream					1	11			33			3	14	2	6									
**13**	Mound					1	12			13	11										2				
**13**	Pit						60	1		105				46	2	2					1		1	1	1
**13**	Upstream		3			2	23	1		81			3	21		7				1	2	1	1		
**14**	Mound		3			3	13			5			3	13		4			1			1			
**14**	Pit					1	8			5				30	1	1									
**14**	Upstream					6	16			2			2	11			1				2	1	1		
**15**	Mound		1			6	23			20			4	9		1									

Nest	Zone	46	47	48	49	50	51	52	53	54	55	56	57	58	59	60	61	62	63	64	65	66	67	68	69
**1**	Mound		2	8	1					3		1												3	52
**1**	Pit			1	1					1															27
**1**	Upstream	1		8	1					2														1	19
**2**	Mound			4	1					1												1		1	24
**2**	Pit		1	2	1					1															27
**2**	Upstream		3	1	1					1														2	4
**3**	Mound			3	1								1											4	20
**3**	Pit		2	2	1					1															6
**3**	Upstream	1	1	27	1									1						1				5	21
**4**	Mound				1																			1	16
**4**	Pit	1		3	1					1	1		1	1						1				1	6
**4**	Upstream		1	1	1					2			3												18
**5**	Mound		1		1					2															16
**5**	Pit		1		1																				4
**5**	Upstream	1		5	1									1										2	23
**6**	Mound	1	1	12	1					3															9
**6**	Pit		5	3	1					5														2	4
**6**	Upstream		1	6	1					3														2	7
**7**	Mound	1	1		1								1												13
**7**	Pit			8	1					1			1											4	12
**7**	Upstream		1	2	1				1															1	6
**8**	Mound		1	1	1										1										17
**8**	Pit		2	7	1									1										1	15
**8**	Upstream			2	1																				13
**9**	Mound			3	1																				14
**9**	Pit		1		1					1															1
**9**	Upstream		1	6	1																			4	9
**10**	Mound		2	10	1			1	1	4												2			26
**10**	Pit	1	1	11	1					8														2	22
**10**	Upstream	1	1	9	1					4				2										1	27
**11**	Mound		2	13	1				1					3								3			34
**11**	Pit		3	10	1					2														1	30
**11**	Upstream		1	16	1																			5	23
**12**	Mound			1	1																	1		7	13
**12**	Pit		2	1	1					3			1												9
**12**	Upstream	1	2	9	1					2														5	15
**13**	Mound			2	1					1						1									35
**13**	Pit			6	1			1		6												1		3	54
**13**	Upstream		2	13	1				1	3				1										5	32
**14**	Mound			5	1			3		2			1						1					14	11
**14**	Pit		1	3	1			3																1	16
**14**	Upstream			2	1																			5	7
**15**	Mound	1		7	1						1			2	1									7	17

Continued on next page

S 1 – *Continued from previous page*

Nest	Zone	Num. of ind.	Num. of taxa	1	2	3	4	5	6	7	8	9	10	11	12	13	14	15	16	17	18	19	20	21
**15**	Pit	90	15	1								1				14	1							8
**15**	Upstream	125	23	16			1				3	4				15	5							8
**16**	Mound	219	17				2				14	29				15	99							13
**16**	Pit	34	10								1	3				5	1							1
**16**	Upstream	285	27	9		2	3		2		3	21				33	61							35
**17**	Mound	189	17				3				4	19				21	102							8
**17**	Pit	169	19	3							2	10				14	9							28
**17**	Upstream	166	21	8							1	7		1		24	48							11
**18**	Mound	158	18				4					15		1		19	45							16
**18**	Pit	247	23	2			1		1			6				44	11							54
**18**	Upstream	263	26	3			4				1	9				26	40							56
**19**	Mound	226	12				9				1	27				6	130							19
**19**	Pit	39	10				1									4								3
**19**	Upstream	151	23	6				2			1	9				24	18							18
**20**	Mound	239	29	29					5		3	9		1		20	36					1		19
**20**	Pit	157	22	1							1	2				7	4							9
**20**	Upstream	186	22	2							2	15	1			35	56							7
**21**	Mound	312	29	2			1		1		3	7		5		29	141				2			31
**21**	Pit	94	19	1						1	1	2				4	4			1				5
**21**	Upstream	141	27	8		1	1				2	8		2		18	27							15
**22**	Mound	210	21	5			5				2	24				47	46							20
**22**	Pit	116	18	8								4			1	20	3			1				5
**22**	Upstream	195	24	2			3				3	15				32	50							23
**23**	Mound	99	15				1				1	8				10	31							21
**23**	Pit	47	12	1												1	1							3
**23**	Upstream	292	28	8			3		2		2	21				34	42					1		23
**24**	Mound	265	24	7				1	4		3	23		3		54	5							21
**24**	Pit	182	20	1				1				1				21	2							12
**24**	Upstream	175	19						1			9		2		43	2							9
**25**	Mound	201	17				2					7				40	94							13
**25**	Pit	92	19	2			1					7		1		10	5							6
**25**	Upstream	134	16	7			2					5				9	50							5
**26**	Mound	105	13				1					2				25	25							21
**26**	Pit	71	14	2								1				10	2							9
**26**	Upstream	132	14	2			1				1	11				24	41							9
**27**	Mound	242	27	2			7		1		3	15				15	52							37
**27**	Pit	76	13	2							2	3				6	7							23
**27**	Upstream	327	23	14			2			1	3	14				6	81				1	1		78
**28**	Mound	169	18				2	1	1		2	13				25	50							14
**28**	Pit	107	17	1					1			7		2		14	6							2
**28**	Upstream	107	22	4			1	1			1	4				15	16							5
**29**	Mound	153	22	2								15				12	6				1			23
**29**	Pit	236	27	19			1		2		1	15				13	7		1					28
**29**	Upstream	187	25	17			1	1	3			8				42	9				1			7
**30**	Mound	267	16		1		6				4	15				12	172							24
**30**	Pit	120	16	3							1	5				9							1	19
**30**	Upstream	148	23	11			1		2		2	11		1		10	24							15

Nest	Zone	22	23	24	25	26	27	27	29	30	31	32	33	34	35	36	37	38	39	40	41	42	43	44	45	46
**15**	Pit						8			10				28	2	1					2		1			
**15**	Upstream		1			3	8			13			1	19		2			1		1					1
**16**	Mound					1	11						3	4		2				1		2				
**16**	Pit						2							4												
**16**	Upstream					7	40	1		3		1	6	20	1	8			2			1				1
**17**	Mound		2			2	11						1	2		1					1					
**17**	Pit					2	29	1		17			4	15		3										
**17**	Upstream		1				21	1		13			1	5		1					2					
**18**	Mound		1			2	28			2			4	4		1			1						1	
**18**	Pit					6	29		1	17	1		3	23		2					2		1			
**18**	Upstream					2	19			8	1		6	40	1	9				2	1	1	1			
**19**	Mound			1		1	16						2													
**19**	Pit						9			4			1	12												
**19**	Upstream		1			1	25			8			2	14		1			1						1	1
**20**	Mound		2			3	9			26	1		3	15		2			1		2		1		1	
**20**	Pit		1			1	22			26		1	1	33		3							3			1
**20**	Upstream		1			4	9	1		8			2	8		1					3					
**21**	Mound			2	1	6	25			3	1		7	13	1	2			1		1	1				
**21**	Pit						10			4			1	32								2	1			
**21**	Upstream					2	8						6	12		1					1					1
**22**	Mound		2			1	18			1			6	8	2	2			2	1						
**22**	Pit					2	18			10				9		1										
**22**	Upstream		2	1		2	21			2			2	17	1	1										1
**23**	Mound			1			9			1				2		1				1		1				
**23**	Pit						3			5				23		1					1					
**23**	Upstream		3			2	30			19			2	23	1	5					2					1
**24**	Mound					3	24			23			8	16	1	1					3					
**24**	Pit						30			24			1	54	1	3					1		2			
**24**	Upstream					13	20			23			8	9		1					3					
**25**	Mound		1	1		2	4						2	2							1	1				
**25**	Pit					2	13			2				21							1		1			
**25**	Upstream					4	2			1				7		1										
**26**	Mound						2			1				4								2				
**26**	Pit						7			5				22	1	2										
**26**	Upstream						8			4				7		2										
**27**	Mound	2		1	1	3	36			4		1	5	14		6					2	1			1	3
**27**	Pit						5			2				7							2					
**27**	Upstream					4	32			3			1	36		8				1	2	1				
**28**	Mound					4	12			1				4	1						2	2				
**28**	Pit					1	16			3			1	33		2										
**28**	Upstream						15			3			2	8		1					1	2	1			
**29**	Mound		2			8	13	1		3			3	10									2			
**29**	Pit		2			7	6			35			2	19		2					1		3		2	
**29**	Upstream					3	13			9			4	13	1	3					4					
**30**	Mound						10						3	1						1		2				
**30**	Pit					3	15			4				28		2					1					
**30**	Upstream					3	26			3			1	13		3					3	1				

Nest	Zone	47	48	49	50	51	52	53	54	55	56	57	58	59	60	61	62	63	64	65	66	67	68	69
**15**	Pit	2					1																	10
**15**	Upstream			1			2		2												1		5	12
**16**	Mound		2	1																			1	19
**16**	Pit		1	1																				15
**16**	Upstream		5	1			2		1														4	12
**17**	Mound		1	1					1															9
**17**	Pit		7	1					4												2		5	13
**17**	Upstream		7	1					2					1									5	5
**18**	Mound		4									1												9
**18**	Pit	3	21	1					2												4			12
**18**	Upstream	1	14	1					2												1		7	7
**19**	Mound			1																				13
**19**	Pit		2	1																				2
**19**	Upstream		3	1					2			1											3	8
**20**	Mound		13	1			2		3				3								5		2	21
**20**	Pit		8	1			2		2												2			26
**20**	Upstream		2	1					2				1										5	20
**21**	Mound		13	1					3				1										3	5
**21**	Pit		2	1					3											1				18
**21**	Upstream	2	7	1		1	1		3			1								1		1	2	8
**22**	Mound			1								1											3	13
**22**	Pit		6	1					1												1		1	24
**22**	Upstream		4	1					1							1					1		2	7
**23**	Mound			1																				10
**23**	Pit			1					2															5
**23**	Upstream	2	39	1					4			4					1				2		1	14
**24**	Mound		7	1					2			1									7		1	46
**24**	Pit			1					1	1		1								1				23
**24**	Upstream		9	1								3	1								7			11
**25**	Mound			1					1			2												27
**25**	Pit		5	1			1		3														2	8
**25**	Upstream			1			3		2														1	34
**26**	Mound	1		1					2															18
**26**	Pit		1	1										1										7
**26**	Upstream			1																			3	18
**27**	Mound		9	1					2														1	17
**27**	Pit		1	1																				15
**27**	Upstream		4				1		2															31
**28**	Mound			1					1															33
**28**	Pit		3	1			1																	13
**28**	Upstream	2		1					1			2											5	16
**29**	Mound		28	1	1				1					1							4		4	12
**29**	Pit		28	1			1		7								1				5		4	23
**29**	Upstream		28	1					1			1		1							3		1	12
**30**	Mound			1					1			1												13
**30**	Pit		11	1													1							16
**30**	Upstream		4	1					1												1		2	9
