# Supplementary material for: Sea lamprey nests promote the diversity of benthic macroinvertebrate assemblages
Source: PLoS One. 2022 Dec 15;17(12):e0274719. doi: 10.1371/journal.pone.0274719 (PMC9754182; doi:10.1371/journal.pone.0274719)
Supplement: Attachment — Submitted filename: PONE-D-22-24495_reviewer (1).pdf [file pone.0274719.s004.pdf]

PLOS ONE

Sea lamprey nests promote the diversity of benthic macroinvertebrate assemblages

--Manuscript Draft--

**Manuscript Number:**	PONE-D-22-24495
**Article Type:**	Research Article
**Full Title:**	Sea lamprey nests promote the diversity of benthic macroinvertebrate assemblages
**Short Title:**	Sea lamprey promotes benthic macroinvertebrate diversity
**Corresponding Author:**	Marius Dhamelincourt<br>UMR ECOBIOP (INRAE/UPPA)<br>Saint-Pée-sur-Nivelle, Aquitaine FRANCE
**Keywords:**	communities; lamprey; spawning; freshwater; substrate
**Abstract:**	The habitat heterogeneity hypothesis states that increased habitat heterogeneity promotes species diversity through increased availability of ecological niches. We aimed at describing the local-scale effects of nests of the sea lamprey (<i>Petromyzon marinus</i> L.) as ecosystem engineer on macroinvertebrate assemblages. We hypothesized that increased streambed physical heterogeneity caused by sea lamprey spawning would modify invertebrate assemblages and specific biologic traits and promote reach-scale diversity. We sampled thirty lamprey nests of the Nive River in three zones: the unmodified riverbed (control) and zones corresponding to the nest: the area excavated (pit) and the downstream accumulation of pebbles and cobbles (mound). The increased habitat heterogeneity created by lamprey was traduced by biological heterogeneity with a reduced density of invertebrates (1160 to 6540 individuals per m² in control, 680 to 6460 individuals per m² in pit and 1980 to 6240 individuals per m² in mound) and number of taxa (23.5 ± 3.9 taxa for control, 18.6 ± 3.9 taxa in pit and 21.2 ± 4.5 taxa for mound) in the pit compared to other zones. However the overall taxa diversity in nest increased with 82 ± 14 taxa compared to the 69 ± 8 taxa estimated in control zone. Diversity indices were consistent with the previous results indicating a loss of α diversity in pit but a higher β diversity between a pit and a mound than between two control zones, especially considering Morisita index accounting for taxa abundance. Trait analysis showed high functional diversity within zones with a reduced proportion of collectors, scrapers, shredders, litter/mud preference and small invertebrates in mound, while the proportion of "slabs, blocks, stones and pebbles" preference and largest invertebrates increased. Pit presented the opposite trend, while control had globally intermediate trait proportions. Our results highlight important effects on species and functional diversity due to habitat heterogeneity created by a nest-building species, what can ultimately influence food webs and nutrient processes in river ecosystems.
**Order of Authors:**	Marius Dhamelincourt
	Jacques Rives
	Marie Pons
	Aitor Larrañaga
	Cédric Tentelier
	Arturo Elosegi
**Additional Information:**	
**Question**	**Response**
**Financial Disclosure**<br><br>Enter a financial disclosure statement that describes the sources of funding for the work included in this submission. Review the submission guidelines for detailed	Functioning was financed by Pôle Gestion des Migrateurs Amphihalins dans leur Environnement (https://www6.rennes.inrae.fr/u3e/PRESENTATION/Organisation/Pole-MIAME). M.D. PhDs was financed by University of Pau and Pays de l'Adour (https://www.univ-pau.fr/fr/index.html) and UPV/EHU (https://www.ehu.eus/es/home). Field work used resources from the IE ECP Experimental Facility of the UMR Ecobiop (https://www6.bordeaux-aquitaine.inrae.fr/ie-ecp-ecobiop).

Powered by Editorial Manager® and ProduXion Manager® from Aries Systems Corporation

requirements. View published research articles from *PLOS ONE* for specific examples.<br><br>This statement is required for submission and **will appear in the published article** if the submission is accepted. Please make sure it is accurate.<br><br>**Unfunded studies**<br>Enter: *The author(s) received no specific funding for this work.*<br><br>**Funded studies**<br>Enter a statement with the following details:<br>• Initials of the authors who received each award<br>• Grant numbers awarded to each author<br>• The full name of each funder<br>• URL of each funder website<br>• Did the sponsors or funders play any role in the study design, data collection and analysis, decision to publish, or preparation of the manuscript?<br>• **NO** - Include this sentence at the end of your statement: *The funders had no role in study design, data collection and analysis, decision to publish, or preparation of the manuscript.*<br>• **YES** - Specify the role(s) played.<br><br>* typeset	The funders had no role in study design, data collection and analysis, decision to publish, or preparation of the manuscript.
**Competing Interests**<br><br>Use the instructions below to enter a competing interest statement for this submission. On behalf of all authors, disclose any competing interests that could be perceived to bias this work—acknowledging all financial support and any other relevant financial or non-financial competing interests.<br><br>This statement is required for submission and **will appear in the published article** if the submission is accepted. Please make sure it is accurate and that any funding sources listed in your Funding Information later in the submission form are also declared in your Financial Disclosure	The authors have declared that no competing interests exist.

statement.<br><br>View published research articles from *PLOS ONE* for specific examples.<br><br>**NO authors have competing interests**<br><br>Enter: *The authors have declared that no competing interests exist.*<br><br>**Authors with competing interests**<br><br>Enter competing interest details beginning with this statement:<br><br>*I have read the journal's policy and the authors of this manuscript have the following competing interests: [insert competing interests here]*<br><br>* typeset	
**Ethics Statement**<br><br>Enter an ethics statement for this submission. This statement is required if the study involved:<br><br>• Human participants<br>• Human specimens or tissue<br>• Vertebrate animals or cephalopods<br>• Vertebrate embryos or tissues<br>• Field research<br><br>Write "N/A" if the submission does not require an ethics statement.<br><br>General guidance is provided below. Consult the submission guidelines for detailed instructions. **Make sure that all information entered here is included in the Methods section of the manuscript.**	N/A

Powered by Editorial Manager® and ProduXion Manager® from Aries Systems Corporation

**Format for specific study types**<br><br>**Human Subject Research (involving human participants and/or tissue)**<br>• Give the name of the institutional review board or ethics committee that approved the study<br>• Include the approval number and/or a statement indicating approval of this research<br>• Indicate the form of consent obtained (written/oral) or the reason that consent was not obtained (e.g. the data were analyzed anonymously)<br><br>**Animal Research (involving vertebrate animals, embryos or tissues)**<br>• Provide the name of the Institutional Animal Care and Use Committee (IACUC) or other relevant ethics board that reviewed the study protocol, and indicate whether they approved this research or granted a formal waiver of ethical approval<br>• Include an approval number if one was obtained<br>• If the study involved *non-human primates*, add *additional details* about animal welfare and steps taken to ameliorate suffering<br>• If anesthesia, euthanasia, or any kind of animal sacrifice is part of the study, include briefly which substances and/or methods were applied<br><br>**Field Research**<br><br>Include the following details if this study involves the collection of plant, animal, or other materials from a natural setting:<br>• Field permit number<br>• Name of the institution or relevant body that granted permission	
**Data Availability**<br><br>Authors are required to make all data underlying the findings described fully available, without restriction, and from the time of publication. PLOS allows rare exceptions to address legal and ethical concerns. See the PLOS Data Policy and FAQ for detailed information.	Yes - all data are fully available without restriction

A Data Availability Statement describing where the data can be found is required at submission. Your answers to this question constitute the Data Availability Statement and **will be published in the article**, if accepted.<br><br>**Important:** Stating 'data available on request from the author' is not sufficient. If your data are only available upon request, select 'No' for the first question and explain your exceptional situation in the text box.<br><br>Do the authors confirm that all data underlying the findings described in their manuscript are fully available without restriction?	
**Describe where the data may be found in full sentences. If you are copying our sample text, replace any instances of XXX with the appropriate details.**<br><br>• If the data are **held or will be held in a public repository**, include URLs, accession numbers or DOIs. If this information will only be available after acceptance, indicate this by ticking the box below. For example: *All XXX files are available from the XXX database (accession number(s) XXX, XXX.*).<br>• If the data are all contained **within the manuscript and/or Supporting Information files**, enter the following: *All relevant data are within the manuscript and its Supporting Information files.*<br>• If neither of these applies but you are able to provide **details of access elsewhere**, with or without limitations, please do so. For example:<br><br>*Data cannot be shared publicly because of [XXX]. Data are available from the XXX Institutional Data Access / Ethics Committee (contact via XXX) for researchers who meet the criteria for access to confidential data.*<br><br>*The data underlying the results presented in the study are available from (include the name of the third party*	Data and code to run the analysis are available in the INRAE dataverse repository at https://doi.org/10.15454/BEHOQK,<br>under the name "Sea lamprey nests promote the diversity of benthic macroinvertebrate assemblages"

Powered by Editorial Manager® and ProduXion Manager® from Aries Systems Corporation

and contact information or URL).<br>• This text is appropriate if the data are owned by a third party and authors do not have permission to share the data.<br><br>* typeset	
Additional data availability information:	

Manuscript

Click here to access/download;Manuscript;Manuscript_formatted.pdf

Sea lamprey nests promote the diversity of benthic macroinvertebrate assemblages

Marius Dhamelincourt[1,2,*], Jacques Rives[1,2], Marie Pons[3], Aitor Larrañaga[4], Cédric Tentelier[1,2], and Arturo Elosegi[4]

[1]Université de Pau et des Pays de l'Adour, E2S UPPA, INRAE, ECOBIOP, Saint-Pée-sur-Nivelle, France

[2]Pôle Gestion des Migrateurs Amphihalins dans leur Environnement, OFB, INRAE, Agrocampus Ouest, Univ Pau & Pays Adour/ E2S UPPA, Pau, France

[3]Bureau d'études Aquabio, Route de Créon, 33750 Saint-Germain-du-Puch

[4]Department of Plant Biology and Ecology, University of the Basque Country (UPV/EHU), Bilbao, Spain

[*]Corresponding author; E-mail: marius.dhamelincourt@inrae.fr

September 1, 2022

Keywords: communities, lamprey, spawning, freshwater, substrate

Abstract

The habitat heterogeneity hypothesis states that increased habitat heterogeneity promotes species diversity through increased availability of ecological niches. We aimed at describing

the local-scale effects of nests of the sea lamprey (*Petromyzon marinus* L.) as ecosystem engineer on macroinvertebrate assemblages. We hypothesized that increased streambed physical heterogeneity caused by sea lamprey spawning would modify invertebrate assemblages and specific biologic traits and promote reach-scale diversity. We sampled thirty lamprey nests of the Nive River in three zones: the unmodified riverbed (control) and zones corresponding to the nest: the area excavated (pit) and the downstream accumulation of pebbles and cobbles (mound). The increased habitat heterogeneity created by lamprey was traduced by biological heterogeneity with a reduced density of invertebrates (1160 to 6540 individuals per m^2 in control, 680 to 6460 individuals per m^2 in pit and 1980 to 6240 individuals per m^2 in mound) and number of taxa (23.5 $\pm$ 3.9 taxa for control, 18.6 $\pm$ 3.9 taxa in pit and 21.2 $\pm$ 4.5 taxa for mound) in the pit compared to other zones. However the overall taxa diversity in nest increased with 82 $\pm$ 14 taxa compared to the 69 $\pm$ 8 taxa estimated in control zone. Diversity indices were consistent with the previous results indicating a loss of α diversity in pit but a higher β diversity between a pit and a mound than between two control zones, especially considering Morisita index accounting for taxa abundance. Trait analysis showed high functional diversity within zones with a reduced proportion of collectors, scrapers, shredders, litter/mud preference and small invertebrates in mound, while the proportion of "slabs, blocks, stones and pebbles" preference and largest invertebrates increased. Pit presented the opposite trend, while control had globally intermediate trait proportions. Our results highlight important effects on species and functional diversity due to habitat heterogeneity created by a nest-building species, what can ultimately influence food webs and nutrient processes in river ecosystems.

Introduction

Habitat heterogeneity has been widely recognized as an important factor structuring biological assemblages [1, 2]. The habitat heterogeneity hypothesis [3] states that increased habitat

heterogeneity promotes species diversity thanks to a greater number of available ecological niches. Li and Reynolds [4] defined heterogeneity in an operational way as "the complexity and/or variability of a system property in space and/or time". Heterogeneity can be considered at different scales, and depending on the scale considered, it has contrasted effects on ecological processes [5]. In the case of stream invertebrates, substrate heterogeneity can affect food distribution [6] and thus condition their local-scale (i.e. nest-scale) feeding and movements [7], whereas movements at larger scales can respond to drift if there are no available interstices or substrate for attachment.

In aquatic ecosystems, heterogeneity analyzes are often used to explain macroinvertebrate assemblages. Beisel et al. [8] found that the number of taxa in 0.5 to 4 m radius zones was higher in a heterogeneous environment with diverse substrate and an elevated patchiness. Furthermore, the same study found that homogeneous environments are more prone to the dominance of few taxa, probably due to the lack of competition with taxa from neighbouring patches. At the sample scale, heterogeneous substrate hosts more diverse assemblages as found by Boyero [9] for 225 cm^2 samples. Substrate appears to be a key driver of macroinvertebrate assemblage structure followed by current velocity and depth [10]. However, increased heterogeneity does not necessarily enhance macroinvertebrate diversity, at least in stream restoration projects [11].

Factors influencing heterogeneity include abiotic and biotic processes, such as the interaction between hydraulics and the substrate, or the effects of engineer species [12]. Together, they affect habitat heterogeneity, which in turn can affect ecosystem functioning [6]. Substrate disturbance by foraging fish is another cause of substrate heterogeneity, which may destabilize the substrate and cause an increased bedload transport during subsequent flood events [13].

Fish nesting, especially in species such as salmonids, which move large volumes of sediment and alter local bed morphology, is a biotic process that can increase riverbed

substrate heterogeneity [14]. For migratory species with high nest density in the spawning grounds, the ecological effects can be important and cascade to affect biological assemblages and ecosystem processes. For instance, Moore and Schindler [15, 16] found that bed disturbance by salmon in spawning grounds caused severe seasonal declines in periphyton and benthic invertebrate abundance, with no recovery within the same season. However, other species such as chubs of the genus *Nocomis* build nests that promote macroinvertebrate density and even allow some taxa to persist in degraded streams [17]. Migratory species likely to affect substrate heterogeneity and the assemblages include the sea lamprey (*Petromyzon marinus* L.), which builds 40-220 cm-wide nests [18, 19, 20] in late spring [21]. Both male and female lamprey remove cobbles with their mouth and release them downstream so that the resulting nest consists of a pit carpeted with sand or gravel, followed by a mound of pebbles and cobbles. This structural heterogeneity might have functional consequences, as the density of some taxa such as Hydropsychidae, Philopotamidae and Heptageniidae can increase up to tenfolds in mounds [22] in an oligotrophic stream. Another study [23] found an increased Simuliidae density in areas with disturbed substrate and added sea lamprey carcasses. These results suggest an effect of sea lamprey nesting activity on macroinvertebrate assemblages, with potential functional consequences.

Here, we aimed at describing the local-scale effects of sea lamprey as ecosystem engineer on macroinvertebrate assemblages at the nest. We expect the taxonomic and functional diversity on the nest as a whole (pit+mound) to be higher than on undisturbed substrate. We hypothesized that increased streambed physical heterogeneity caused by sea lamprey nesting would promote invertebrate diversity and specific biologic traits. Pits, with deeper and more lentic conditions than unmodified riverbed (control) would attract lentophilic and fine sediment fauna, whereas the shallower and more lotic mounds would attract taxa with opposite preferences. In addition, nests with more heterogeneous current and depth may host more diverse communities. To test our hypotheses, we first assessed the variability of depth and

current velocity on pit, mound and control as these variables are among the most likely to explain differences in macroinvertebrate assemblages [10]. Then, we compared the density, taxa richness and diversity of macroinvertebrates across nest zones. Finally, we studied ecological traits describing food type, mode of alimentation, substrate preference and body size, to infer the effects of nests on invertebrate functional heterogeneity.

Methods

Study site and experimental period

The study took place in the Nive River, a 79 km long river situated in Northern Basque Country, France, and draining a basin of 1030 km². The selected section corresponds to a reach of 400 m long, located in Saint-Martin-d'Arrossa (43° 14' 34.926" N, 1° 18' 27.305" W) and bypassed by a hydropower scheme. It is mainly composed of riffles and runs suitable for sea lamprey nesting, as shown by the 46 nests found on a previous survey [24]. Macroinvertebrate samples were collected on July 7 2020, at the end of the reproductive season, to avoid disturbing sea lamprey spawning. No individuals were observed in the two weeks prior to sampling, a period of colonization short enough to limit the risk of flooding but sufficient to allow for stability of local invertebrate assemblages [25].

Macroinvertebrate samples and nest characteristics

We studied 30 completed nests where no lamprey was observed. For each nest, we defined three zones: the **control zone**, 20 to 50 cm upstream from the upstream verge of the nest pit, indicated the initial conditions of the habitat chosen by the lamprey to build their nest. The second zone was the **pit**, delimited as the area excavated by the lamprey, where water was deeper and the substrate finer than on the control. The third zone was the **mound**, corresponding to the downstream accumulation of pebbles and cobbles. For all three zones

current velocity (± 1 cm/s) was measured at 5 cm from the bottom (using a magnetic flowmeter "FLO-MATE 2000") and water depth (± 1 cm) was measured with a Vernier gauge. Transversal (perpendicular to the current) and longitudinal (parallel to the current) diameters of the pit were measured using a measuring tape (± 1 cm).

Macroinvertebrates were sampled using a Surber net (1/20 m^2, 0,5 mm mesh) by stirring the entire top layer of the sediment. Three samples, one per zone, were collected on each nest. After collection the samples were stored in 70° ethanol up to the determination. Most invertebrates were identified following AFNOR standard XP T 90-388 at the genus level except for Oligochaeta (subclass), Diptera (family) and some individuals for whom genus determination was not certain (determined at the family level). Macroinvertebrate traits were determined following Tachet et al. [26] and expressed as the frequency of individuals belonging to a certain trait over the total number of individuals in the sample. We selected food type, mode of alimentation, substrate preference and body size as these traits are likely to vary according to the physical characteristics of the nests. Food type and mode of alimentation directly reflect the resources provided by a given environment. With different current and substrate characteristics those traits should vary between zones. As substrate will differ, substrate preferences may reflect the sorting made by spawners between zones, if the scale of the nest has an impact on macroinvertebrate spatial location. Finally, body size emphasizes the carrying capacity for smaller or bigger invertebrates, which completes the substrate preference.

Invertebrate diversity

We used several α and β indices to measure the effects of lamprey nests on the diversity of macroinvertebrate assemblages. For α indices we calculated the indices in each zone to compare the pit and the mound with the control. First, we measured the species richness with the specpool function of the vegan package [27, version 2.5-7] using a Chao1 Eq [28]. Then, Shannon α diversity index [29, Eq 1] indicated how diverse the taxa in each zone are,

increasing with the increase of evenness and taxa richness in a sample. Shannon index was completed with Pielou index [30, Eq 2] to specifically analyze the evenness of the assemblages. These indices were transformed into Log Response Ratio (LRR), corresponding to the effect size of the diversity differences between nest (pit/mound) zones and control zones (Eq 3 and Fig 4) where the "index modified zone" corresponds either to the index of a pit or to the index of a mound. Indices were calculated for each pit and each mound and compared to the mean of Shannon and Pielou indices obtained for the control zones ("index control zone"). Behind this analysis, we compared how a given index (Shannon or Pielou) evolved on a pit or on a mound - thus reflecting a disturbed habitat - compared to the average value of these indices in the section considered.

$$Shannon\ index\left(H'\right) = -\sum_{i=1}^{S} p_i \ln p_i \quad (1)$$

Where p_i is the proportion of a taxon i on the total number of individuals N and S is the total number of taxa in the sample.

$$Pielou\ evenness\ index\left(J'\right) = \frac{H'}{H'_{\max}} \quad (2)$$

Where H'is the Shannon diversity index and H'_{max} is the maximum possible value of H'(if every species was equally likely): $H'_{\max} = -\sum_{i=1}^{S} \frac{1}{S} \ln \frac{1}{S}$ and S being the total number of taxa in the sample.

$$Log\ Response\ Ratio = \ln\left(\frac{index\ modified\ zone}{index\ control\ zone}\right) \quad (3)$$

Following this α diversity overview we studied several β diversity aspects to compare nest and control. For each index we calculated all the pairwise differences between pit and mound and between two control zones. Firstly, we calculated Sorensen dissimilarity index [31, Eq 4] which includes both replacement and nestedness and corresponds to a global indicator of taxa diversity. Then we calculated Simpson β diversity index to study taxa replacement [31, Eq 5].

The second component of the Sorensen index, the taxa nestedness, was obtained by computing the difference between Sorensen and Simpson indices [32]. Finally, to test the diversity considering taxa abundance and not solely their occurrence, we calculated the Morisita dissimilarity index [33, Eq 6]. As for the α diversity indices the β indices were transformed into Log Response Ratio (Eq 3 and Fig 5) where the "index modified zone" corresponds to a pairwise index between a pit and a mound and the "index control zone" to a pairwise index between two controls. This LRR indicates if the diversity difference between two locations of a nest (always a pit versus a mound) differs from the same comparison made between two controls, thus reflecting a difference in taxa heterogeneity. To avoid a nest effect we did not compare the taxa of a pit versus the mound of the same nest and therefore we randomly selected the pit and the mound to be compared in each index value.

$$Sorensen\ \beta\ diversity\ index = \frac{(b+c)}{(2a+b+c)} \tag{4}$$

Where a is the number of common taxa between two sites, b the number of taxa exclusive to a site, and c the number of taxa exclusive to the other site.

$$Simpson\ \beta\ diversity\ index = \frac{\min(b,c)}{\min(b,c)+a} \tag{5}$$

Where min (b, c) is the lowest number of exclusive taxa between two sites, b being the number of taxa exclusive to a site, and c the number of taxa exclusive to the other site; a is the number of common taxa between the two sites.

$$Morisita\ overlap\ index\,(C_D) = \frac{2\sum_{i=1}^{S} x_i y_i}{(D_x+D_y)XY} \tag{6}$$

Where x_i is the number of times taxon i is represented in the total X from one site; y_i is the number of times taxon i is represented in the total Y from another site; D_x and D_y are the Simpson's α diversity index for the x and y sites respectively:

$$D_i = \sum_{i=1}^{S} p_i^2 \tag{7}$$

With S the total number of taxa in the sample and p_i the proportion of a taxon i on the total number of individuals N.

Data analysis

All analyzes were performed using R software version 4.1.0 [34] and a significance level of 0.05.

The significance of differences between nest zones for depth, current, density, diversity, Shannon index and Pielou index was determined using linear mixed model and the *lmer* function of the *lme4* package [35] with a random effect of nest identity. For density and taxa diversity (Fig 2A and B) a pairwise Tukey test was used to check the differences between each zone rather than globally. Trait differences between zones were determined using binomial mixed models with control or mound as reference condition and nest identity as a random effect (function *glmer* of the *lme4* package; Bates et al. [35]. The links between trait and nest characteristics were studied using Redundancy analysis (RDA), a multivariate approach of linear models applied in *vegan* package [27, version 2.5-7] with *rda* function and setting traits as response variables. Traits were implemented after Hellinger transformation to give low weight to traits with low counts and many zeros. Explanatory variables were the five variables describing nest characteristics: transversal and longitudinal diameter, depth, current and depth difference between pit and mound. The latter was calculated assuming nests with higher depth difference could be more heterogeneous with a more important volume and habitat complexity. To test the effect of current and depth on abundance and taxa diversity, a general linear model was used with current and depth as covariates and following a Gaussian distribution.

Results

Nests characteristics

Lamprey nests differed greatly in their dimensions, transversal diameter of the pit averaging 129.2 cm (range, 70-210 cm) and longitudinal diameter 117,4 cm (range, 50-260 cm, Table 1). The pit was the deepest zone (59.2 ± 9.9 cm) followed by the control (51.8 ± 10.1 cm) and the mound (38.3 ± 10.1 cm), and differences were statistically significant considering mixed models p-values (Table 2). The coefficient of variation for depth was similar for all three zones (Table 2). Current velocity ranged from 0 to 65 cm/s (Table 1) and varied among zones inversely to depth (Fig 1), although the coefficient of variation was larger. Again, differences were statistically significant considering mixed models results.

Fig 1. Relationship between depth and current velocity for each nest at each zone. Ellipses correspond to multivariate t-distribution.

Table 1. Summary of the nests characteristics measured in the study; mean ± sd (cv).

	Pit	Mound	Control
Transversal diameter (cm)	129.2 ± 37.2 (0.3)	-	-
Longitudinal diameter (cm)	117.4 ± 45.7 (0.4)	-	-
Depth (cm)	59.2 ± 9.9 (0.2)	38.3 ± 10.1 (0.3)	51.8 ± 10.1 (0.2)
Current (cm/s)	8.8 ± 7.8 (0.9)	43.8 ± 11.6 (0.3)	19.8 ± 11.1 (0.6)
Depth difference (cm)	20.9 ± 4.8 (0.2)		-

Table 2. Mixed model results for analyzes of differences between zones for nest characteristics, density and diversity of macroinvertebrates and α diversity indices, with nest identity as a random effect. With $^{*}P \leq 0.05$, $^{**}P \leq 0.01$, $^{***}P \leq 0.001$.

Variables	*Df*	χ^2	*P*
Depth	2	503.5	<2e-16 ***
Current	2	278.41	<2e-16 ***
Density	2	17.475	1.61e-4 ***
Diversity	2	25.736	2.58e-6 ***
Shannon index (control/pit/mound)	2	12.739	1.71e-3 **
Pielou index (control/pit/mound)	2	6.6937	3.52e-2 *

Macroinvertebrate density and diversity

The density of macroinvertebrates (Fig 2A) ranged from 680 to 6460 individuals per m^2 in pit, from 1980 to 6240 individuals per m^2 in mound and from 1160 to 6540 individuals per m^2 in control. It was significantly lower in pit than in control and mound, but there were no differences between control and mound. The taxa richness (Fig 2B) also did not vary significantly between control (23.5 $\pm$ 3.9 taxa) and mound (21.2 $\pm$ 4.5 taxa) but was higher in these two zones than in pit (18.6 $\pm$ 3.9 taxa). The Venn diagram (Fig 3), describing exclusivity or sharing of taxa, indicates an important taxa overlap with 59 % of the taxa found on all zones. The significant pattern of reduced taxa richness found at the nest scale was not found by summing all the different taxa identified in the thirty samples of each zone and visualizing it in the Venn diagram.

Fig 2. Density of macroinvertebrates (ind/m^2) (A) and number of taxa per sample (B) for the three zones studied.
With ns $P > 0.05$, $^{*}P \leq 0.05$, $^{**}P \leq 0.01$, $^{****}P \leq 0.0001$..

Fig 3. Venn diagram with the distribution of taxa among zones.
The area of each part is proportional to the number of taxa indicated in absolute number and percentage of total taxa richness.

Diversity

The estimate of Chao1 diversity index was 82 ± 14 taxa for nest (pit + mound) and 69 ± 8 taxa for the control zone. In spite of the slight overlap of the standard error of those estimates the species richness seems to be higher in the nest than in the control. Log response ratios of the α diversity indices (Fig 4) showed an overall trend of reduced diversity and equitability for both Shannon and Pielou indices in pit and mound, compared to the average values observed in the control zone. However, only the log response ratio of the Shannon index of the pit is strictly below 0 and indicates a significantly reduced diversity in pit compared to the control zone. The log response ratios of β diversity indices (Fig 5) showed a higher overall β diversity between a pit and a mound than between two control zones. However the log response ratios were highly variable and none were significantly different from 0. Nevertheless the higher Morisita index ratio compared to other indices seems to indicate a differentiation made by taxa abundance rather than by replacement or nestedness.

Fig 4. Log response ratios of Shannon and Pielou α diversity indices for each pit and each mound compared to the mean of Shannon and Pielou indices obtained for the control zones. Dots correspond to the mean and error bars correspond to the standard deviation.

Fig 5. Log response ratios of β diversity indices of all mound and pit pairwise indices, each compared to a pairwise control index among all possible control pairwise comparisons (control zone randomly selected, not directly upstream from the pit and mound considered).
Dots correspond to the mean and error bars correspond to the standard deviation.

Trait analysis

The traits studied tended to be similar in mound and control but different in pit (Table 3 and Figs 6 - 8). Considering alimentation traits (Fig 6), the proportion of collectors and scrapers was lower in the mound than in the pit and the proportion of filterers was lower in pit than either in mound or control. Predators were more present in mound than in pit and control, whereas shredders were less abundant in mound than in pit and control. However, food traits, related to alimentation aspects, did not highlighted significant differences. Substrate preference analysis (Fig 7) showed a similar pattern for litter and mud with higher proportion in pit than in mound. Preference for gravel and sand was more important in pit, followed by control and then mound. Finally, preference for slabs, blocks, stones and pebbles was highest in mound and lowest in pit. The smallest size category (Fig 8) was more represented inside the pit, followed by control and mound, whereas larger size categories followed the inverse pattern.

Table 3. Binomial mixed models results comparing frequency of selected traits between zones with nest identity as a random effect. With $^{*}P \leq 0.05$, $^{***}P \leq 0.001$.

	Zone	*Df*	*Z value*	*P*
Food	Mound (reference: control)	2	-1.016	0.31
	Pit (reference: control)	2	-0.614	0.539
	Mound (reference: pit)	2	-0.401	0.688
Alimentation	Mound (reference: control)	2	1.877	6.05e-2
	Pit (reference: control)	2	-2.201	2.77e-2 *
	Mound (reference: pit)	2	4.063	4.84e-5 ***
Substrate	Mound (reference: control)	2	-6.918	4.57e-12 ***
	Pit (reference: control)	2	10.779	<2e-16 ***
	Mound (reference: pit)	2	-17.391	<2e-16 ***
Size	Mound (reference: control)	2	5.682	1.33e-08 ***
	Pit (reference: control)	2	-11.63	<2e-16 ***
	Mound (reference: pit)	2	16.71	<2e-16 ***

Fig 6. Median percentage of macroinvertebrate alimentation traits for the three zones studied.

Fig 7. Median percentage of macroinvertebrate substrate preferences for the three zones studied.

Fig 8. Median percentage of macroinvertebrate size traits for the three zones studied.

Relationship between traits and nest characteristics

Redundancy analysis (Fig 9) yielded a significant model ($F = 10.876$, $Pr(>F) = 0.001$), although the only significant term was the current ($F = 46.8357$, $Pr(>F) = 0.001$), depth and depth difference being marginally significant ($F = 2.4535, Pr(>F) = 0.077$ and $F = 2.6966$, $Pr(>F) = 0.077$ respectively). The ellipses indicated a separation of pit from mound and control structured by current and "< *5 mm*", "*scrapers*", "*sand*" and "*gravel*" trait categories belonging to the pit ellipse. Control and mound were not clearly separated but "*5-10 mm*", "*slabs, blocks, stones and pebbles*" and "*filterers*" belong to the mound ellipse. Those results are consistent with the specific trait analyzes (Figs 6 - 8). Depth, if not the most discriminant variable, explained the separation of pit from other zones.

Fig 9. Redundancy analysis between nest characteristics and macroinvertebrate traits. Slabs = *slabs, blocks, stones and pebbles*.

Generalized Linear Models indicated that current affected neither the number of taxa nor the macroinvertebrate density ($tvalue = 0.391$; $Pr(>|t|) = 0.697$ and $tvalue = 1.470$; $Pr(>|t|) = 0.1452$ respectively). Depth influenced negatively the macroinvertebrate density ($tvalue = -2.026$; $Pr(>|t|) = 0.0458$) but not the number of taxa ($tvalue = -0.821$; $Pr(>|t|) = 0.414$).

Discussion

Firstly, our results globally showed that habitat diversity and structural heterogeneity due to lamprey nesting activities modify several aspects of biodiversity. The clear distinction between nest zones regarding depth and riverbed current velocity implied biological heterogeneity, a non trivial result as previously highlighted [11]. The pit had a lower density of invertebrates and a lower number of taxa than the other zones, which did not differ between them, although the number of taxa tended to be higher at the control. Log response ratio of α Shannon index

showed a similar trend concerning the diversity inside the pit. Those results indicate that nest building reduced the local abundance and diversity of macroinvertebrates in the pit, at least during the first weeks after nest digging, which are the most significant from the point of view of the lamprey, as larvae abandon the nest shortly after hatching [36]. Patches of fine substrate such as the pit tend to be less diverse than zones with coarser grain size [37]. Reduced invertebrate abundance and richness have been also reported in nests of the largemouth bass (*Micropterus salmoides*) [38] and the pink salmon (*Oncorhynchus gorbuscha*) [39] during the spawning season, which was attributed to spawning-related disturbance. On the other hand, Hogg et al. [22] reported that the density of invertebrates in sea lamprey nest mounds were twice that found in pits and 77% higher than in control zone. Their results of reduced macroinvertebrate pit abundance are consistent with ours but we did not observe an increased mound density. An explanation for the important density in mounds could be the dominance of the Chironomidae within the river studied, not found for the Nive River (Fig 10 in supporting information). The difference between our results and theirs seems not to derive from differences in macroinvertebrate assemblages, as these were relatively similar between both studies. A significantly higher number of taxa in control than in mound and pit indicates that sea lamprey nests create heterogeneity but do not increase local species diversity.

In a local scale, i.e. the zone, we found for each pit and mound several taxa not present in the control (see Fig 11 in supporting information). Indeed, whereas control is more complex and offers more substrate types with its unsorted grain size - likely to provide suitable habitat for more taxa - [40], mound and pit provide larger areas of some specific characteristics, including substrate. This wider area may potentially allow the establishment of more taxa sharing similar ecological preferences, when these would be less represented and therefore potentially absent or very rare in the control. Considering the nest scale, rare taxa should be found more easily, either in mound or in pit considering their preferences than in control (considering a same sample size). At a larger scale, occurrence of rare species and so species

diversity would be higher in zones with nests than in zones without them.

In addition to increasing the structural and biological heterogeneity, sea lamprey nests seem to shape invertebrate assemblages. Indeed, three traits over the four studied showed differences between nest and the control zones, although the direction of change did not always follow our expectations. In particular, we expected herbivores to increase in mounds, as epilithic algae tend to be favored by shallow water, coarse sediments and moderately fast flow [41, 42], but no significant difference was found globally for alimentation traits, perhaps because of lack of time for algae to grow on overturned cobbles. The increased proportion of collectors in pits was expected, as these are a preferential place for organic matter deposits. Similarly, collectors tend to be more abundant in fine substrate, which collected more detritus [43]. The higher concentration of organic matter might also explain the higher abundance of shredders and scrapers in pit than in mound. Also, according to our expectations, filterers were more represented in the mound, as they are favoured by fast-flowing areas [44], particularly during low flow conditions [45]. Those results reflect the different dynamics of food provided within the nest zones.

Not surprisingly given the differences in substrate among zones, invertebrates also differed in their preferred substrate traits. Macroinvertebrates associated with coarse substrate were especially abundant in mounds, whereas taxa associated with fine substrate were more abundant in pits. It must be noted that sand was almost absent in the study reach apart from lamprey nests and some marginal areas, which suggests that nesting lampreys create patches of habitat for lentophylic species in river stretches where these species could not dwell otherwise.

The macroinvertebrate size traits showed a greater proportion of small invertebrates (< 5 mm) within the pit, including *Esolus sp.*, the most abundant taxon in pit (Fig 10 in supporting information) and *Psychomyia pusilla*. This result could be explained by the carrying capacity of fine substrate. A fine substrate has less shelters than a coarser one. Shelters of fine substrate are more suitable for small size range of macroinvertebrates, whereas bigger

macroinvertebrates are more prone to find shelter in the coarser substrate of the mound or the control. Bêche et al. [46] suggested a better exploitation of refugia for small sized macroinvertebrates. Furthermore, small body size could be a possible resistance trait to disturbances such as riverbed movement [47].

Our results demonstrate that sea lamprey creates physical heterogeneity, which then enhances biological heterogeneity both in assemblage composition and function. The heterogeneity demonstrated in this study was created at the nest scale, but local-scale processes can have a major impact at higher scales such as the reach scale [48]. In the case of lamprey nests in the Nive River, pits average 1.15 m^2 and so the total modified streambed area averages 34.5 m^2, representing 4% of the river streambed in our studied zone. With an average reduction of 30% of macroinvertebrate density in pit (compared to control) a total reduction of 1.2% of density is expected in the studied zone. In a river reacting in a similar way to the Nive, this surface with decreased macroinvertebrate density does not seem to be compensated by a higher density in the mound and so, sea lamprey nests globally decrease the macroinvertebrate density.

Measuring the effect of lamprey nests on reach-scale macroinvertebrate density would require a BACI design [49] including periods and reaches with and without nests. If invertebrates affected by lamprey nesting responded with small-scale movements, the result would be an increase in macroinvertebrate density in control samples of reaches with nests compared to control samples of reaches without nests. If the macroinvertebrates responded drifting, the results would be more complex to interpret and depend on the distribution of nests of sea lamprey. An increase of macroinvertebrate densities could be observed if spawning sites occur upstream. Macroinvertebrates drifting from those nests could colonize and increase the density of downstream control sites. When a spawning ground is located on the upper limit of the area colonized by lampreys, drift will not affect the adjacent area of this spawning ground but may affect downstream sites.

Our results highlight the need for a better understanding of the effects of nest-building species on the river ecosystem. Unlike salmon, whose effects are relatively well-studied [50, 51, 52], the consequences of sea lamprey spawning are still poorly known. Whereas lamprey nests only represent 4% of the river streambed in our study, the occupied area may be much higher, especially for invasive populations of the Great Lakes [53] or below impassable dams, likely to dramatically increase the number of spawners in immediately downstream spawning grounds [24, 54]. Studies exist on the effects of carcasses [55, 23] or substrate modification [22] on a local scale, but not at the river or at the global scales. However, macroinvertebrates are at the bottom of the food chain [56]. Such basal position implies an important relationship with ecosystem components. Firstly, macroinvertebrate assemblages can have an important influence on numerous processes [56], such as nutrient cycles [57, 58, 59], primary productivity [60], decomposition [61, 62, 63], and translocation of materials [64, 65]. Then, they are an important resource for organisms belonging to higher trophic levels such as fish species. Macroinvertebrates are considered as the most important source of food, widespread in all freshwater ecosystems [66].

In our study some trait aspects can provide clues about the effects of sea lamprey on general ecosystem functioning in the river. Increased proportion of shredders in the pit suggests they find suitable conditions such as increased plant debris. On a more global scale a certain amount of material (depending on the nest density and count) could be retained by nests in spite of being carried away by the current, and then consumed by macroinvertebrates. Seeing this impact in late spring when litter input is reduced also indicates that the proportion of shredders should be more important in autumn, where these inputs can represent 73% of annual allochtonous inputs (in temperate deciduous forests; Abelho and Graça [67]). But this retention can occur only if the lamprey nests remain in autumn, without being filled by floods. Changes in streambed complexity created by nests persist during the autumn [22] but a change in hydrological conditions is likely to affect this process. Filterers are another trait category

possibly affecting organic matter processing, being one pathway through which carbon and nutrients are transferred from the water column to the sediments [68]. Filterers being more present in proportion in the mound than in the control suggests that mounds are hotspots of nutrient processing. Therefore, sea lamprey nests could have an important effect on nutrient spiraling in streams, but the persistence of their influence on ecosystem functioning until the next spawning season remains to be assessed. Although nests may persist considering their physical structure, it is likely that the subsidies provided by eggs affect macroinvertebrate colonization dynamics and the distribution of functional groups, as predation was depicted in other species [69, 70]. An assessment of the effects of eggs deposited in the pit on functionality should be done to determine what is their potential contribution to the observed differences.

All these possible different effects imply that the modifications of macroinvertebrate assemblages by sea lampreys highlighted here and in previous studies [22, 23] are likely to modify the general functioning of the rivers. However, it is difficult to determine these consequences precisely due to the complexity inside macroinvertebrate assemblages. Such assessment requires specific studies on the components previously described. We advocate for studies focusing on these effects, important in a context of endangered native populations, likely to disappear in some rivers and consequently modifying their dynamics. In addition, negative impacts of invasive populations through population reduction of their hosts may be mitigated by a positive effect through invertebrate diversity.

Acknowledgements

Functioning was financed by Pôle Gestion des Migrateurs Amphihalins dans leur Environnement. M.D. PhDs was financed by Univ. Pau and Pays Adour and UPV/EHU. Field work used resources from the IE ECP Experimental Facility of the UMR Ecobiop [71].

Author contributions

Conceptualisation: MD, CT and AE; Developing methods: MD, CT, JR and AE; Conducting the research: MD, CT, JR, AE, MP; Data analysis and interpretation: MD helped by AL, AE, CT; Preparation of figures and tables: MD; Writing: MD, critically revised by CT, AL and AE.

Data availability

Data and code to run the analysis are available in the INRAE dataverse repository at https://doi.org/10.15454/BEHOQK, under the name "Sea lamprey nests promote the diversity of benthic macroinvertebrate assemblages"

References

1. Michael J. Cramer and Michael R. Willig. Habitat heterogeneity, species diversity and null models. *Oikos*, 108(2):209–218, 2005. ISSN 1600-0706. doi: 10.1111/j.0030-1299.2005.12944.x.

2. J. Tews, U. Brose, V. Grimm, K. Tielbörger, M. C. Wichmann, M. Schwager, and F. Jeltsch. Animal species diversity driven by habitat heterogeneity/diversity: The importance of keystone structures. *Journal of Biogeography*, 31(1):79–92, 2004. ISSN 1365-2699. doi: 10.1046/j.0305-0270.2003.00994.x.

3. Robert H. MacArthur and John W. MacArthur. On Bird Species Diversity. *Ecology*, 42 (3):594–598, 1961. ISSN 1939-9170. doi: 10.2307/1932254.

4. H. Li and J. F. Reynolds. On Definition and Quantification of Heterogeneity. *Oikos*, 73 (2):280–284, 1995. ISSN 0030-1299. doi: 10.2307/3545921.

5. Adela González-Megías, José María Gómez, and Francisco Sánchez-Piñero. Diversity-habitat heterogeneity relationship at different spatial and temporal scales.

Ecography, 30(1):31–41, 2007. ISSN 1600-0587. doi: 10.1111/j.0906-7590.2007.04867.x.

6. Arturo Elosegi, Lorea Flores, and Joserra Díez. The importance of local processes on river habitat characteristics: a Basque stream case study. *Limnética*, 30(2):0183–196, 2011. ISSN 0213-8409.

7. Lorea Flores, Jose Ramón Díez, Aitor Larrañaga, Cláudia Pascoal, and Arturo Elosegi. Effects of retention site on breakdown of organic matter in a mountain stream. *Freshwater Biology*, 58(6):1267–1278, 2013. ISSN 1365-2427. doi: 10.1111/fwb.12125.

8. Jean-Nicolas Beisel, Philippe Usseglio-Polatera, and Jean-Claude Moreteau. The Spatial Heterogeneity of a River Bottom: A Key Factor Determining Macroinvertebrate Communities. *Hydrobiologia*, 422–423:163–171, April 2000. doi: 10.1023/A:1017094606335.

9. Luz Boyero. The quantification of local substrate heterogeneity in streams and its significance for macroinvertebrate assemblages. *Hydrobiologia*, 499(1):161–168, June 2003. ISSN 1573-5117. doi: 10.1023/A:1026321331092.

10. Jean-Nicolas Beisel, Philippe Usseglio-Polatera, Sandra Thomas, and Jean-Claude Moreteau. Stream community structure in relation to spatial variation: The influence of mesohabitat characteristics. *Hydrobiologia*, 389(1):73–88, December 1998. ISSN 1573-5117. doi: 10.1023/A:1003519429979.

11. Margaret A. Palmer, Holly L. Menninger, and Emily Bernhardt. River restoration, habitat heterogeneity and biodiversity: A failure of theory or practice? *Freshwater Biology*, 55(s1):205–222, 2010. ISSN 1365-2427. doi: 10.1111/j.1365-2427.2009.02372.x.

12. Justin P. Wright, Clive G. Jones, and Alexander S. Flecker. An ecosystem engineer, the beaver, increases species richness at the landscape scale. *Oecologia*, 132(1):96–101, June 2002. ISSN 0029-8549, 1432-1939. doi: 10.1007/s00442-002-0929-1.

13. A. G. Pledger, S. P. Rice, and J. Millett. Bed disturbance via foraging fish increases bedload transport during subsequent high flows and is controlled by fish size and species. *Geomorphology*, 253:83–93, January 2016. ISSN 0169-555X. doi: 10.1016/j.geomorph.2015.09.021.

14. Allen Gottesfeld, Marwan Hassan, and Jon Tunnicliffe. Salmon Bioturbation and Stream Process. *American Fisheries Society Symposium*, 65, January 2008.

15. J. W. Moore and D. E. Schindler. Impacts of Bioturbation by Spawning Salmon on the Community Dynamics and Ecosystem Processes of Alaskan Streams. *AGU Spring Meeting Abstracts*, 21:NB21B–06, May 2005.

16. Jonathan W. Moore and Daniel E. Schindler. Biotic disturbance and benthic community dynamics in salmon-bearing streams. *Journal of Animal Ecology*, 77(2):275–284, 2008. ISSN 1365-2656. doi: 10.1111/j.1365-2656.2007.01336.x.

17. Meredith C. Swartwout, Fiona Keating, and Emmanuel A. Frimpong. A survey of macroinvertebrates colonizing bluehead chub nests in a Virginia stream. *Journal of Freshwater Ecology*, 31(1):147–152, January 2016. ISSN 0270-5060. doi: 10.1080/02705060.2015.1036943.

18. Cory Gardner, Stephen M. Coghlan Jr, and Joseph Zydlewski. Distribution and Abundance of Anadromous Sea Lamprey Spawners in a Fragmented Stream: Current Status and Potential Range Expansion Following Barrier Removal. *Northeastern Naturalist*, 19(1):99–110, March 2012. ISSN 1092-6194, 1938-5307. doi: 10.1656/045.019.0108.

19. R. Sousa, M. J. Araújo, and C. Antunes. Habitat modifications by sea lampreys (*Petromyzon marinus*) during the spawning season: Effects on sediments. *Journal of Applied Ichthyology*, 28(5):766–771, October 2012. ISSN 1439-0426. doi: 10.1111/j.1439-0426.2012.02025.x.

20. Marius Dhamelincourt. Individual and group characteristics affecting nest building in sea lamprey, 2021b.

21. Patrick Grellier. La biologie de la lamproie marine (*Petromyzon marinus* Linne 1758) de la côte atlantique française. *Archimer*, page 18, 1996.

22. Robert S. Hogg, Stephen M. Coghlan, Joseph Zydlewski, and Kevin S. Simon. Anadromous sea lampreys (*Petromyzon marinus*) are ecosystem engineers in a spawning tributary. *Freshwater Biology*, 59(6):1294–1307, 2014. ISSN 1365-2427. doi: 10.1111/fwb.12349.

23. Daniel M. Weaver, Stephen M. Coghlan Jr, and Joseph Zydlewski. Effects of sea lamprey substrate modification and carcass nutrients on macroinvertebrate assemblages in a small Atlantic coastal stream. *Journal of Freshwater Ecology*, 33(1):19–30, January 2018. ISSN 0270-5060. doi: 10.1080/02705060.2017.1417168.

24. Migradour. Suivi de la reproduction de la Lamproie marine sur le bassin de l'Adour - Tranche 1/3, gaves et nives. Technical report, 2010.

25. G. Khalaf and H. Tachet. La dynamique de colonisation des substrats artificiels par les macroinvertebres d'un cours d'eau. *Annales de Limnologie*, 13(2):169–190, 1977. ISSN 0003-4088, 2100-000X. doi: 10.1051/limn/1977003.

26. Henri Tachet, Philippe Richoux, Michel Bournaud, and Philippe Usseglio-Polatera. *Invertébrés d'eau Douce*. Cnrs editions edition, May 2000. ISBN 978-2-271-05745-7.

27. Jari Oksanen, Roeland Kindt, Pierre Legendre, Bob Hara, Gavin Simpson, Peter

Solymos, M Henry, Hank Stevens, Helene Maintainer, and jari Oksanen@oulu. The vegan Package. January 2009.

28. Anne Chao and Chun-Huo Chiu. Species Richness: Estimation and Comparison. In *Wiley StatsRef: Statistics Reference Online*, pages 1–26. American Cancer Society, 2016. ISBN 978-1-118-44511-2. doi: 10.1002/9781118445112.stat03432.pub2.

29. C. E. Shannon. A Mathematical Theory of Communication. *Bell System Technical Journal*, 27(3):379–423, 1948. ISSN 1538-7305. doi: 10.1002/j.1538-7305.1948.tb01338.x.

30. E. C. Pielou. *Ecological Diversity*. Wiley, New York, 1975. ISBN 978-0-471-68925-6.

31. Patricia Koleff, Kevin J. Gaston, and Jack J. Lennon. Measuring beta diversity for presence–absence data. *Journal of Animal Ecology*, 72(3):367–382, 2003. ISSN 1365-2656. doi: 10.1046/j.1365-2656.2003.00710.x.

32. Andrés Baselga. Partitioning the turnover and nestedness components of beta diversity. *Global Ecology and Biogeography*, 19(1):134–143, 2010. ISSN 1466-8238. doi: 10.1111/j.1466-8238.2009.00490.x.

33. Henk Wolda. Similarity indices, sample size and diversity. *Oecologia*, 50(3):296–302, September 1981. ISSN 1432-1939. doi: 10.1007/BF00344966.

34. R Core Team. R: A language and environment for statistical computing. R Foundation for Statistical Computing, 2020.

35. Douglas Bates, Martin Mächler, Ben Bolker, and Steve Walker. Fitting linear mixed-effects models using lme4. *Journal of Statistical Software*, 67(1):1–48, October 2015. ISSN 1548-7660. doi: 10.18637/jss.v067.i01.

36. Amy L. Derosier, Michael L. Jones, and Kim T. Scribner. Dispersal of sea lamprey larvae during early life: Relevance for recruitment dynamics. *Environmental Biology of*

Fishes, 78(3):271–284, March 2007. ISSN 1573-5133. doi: 10.1007/s10641-006-9095-3.

37. Xuehua Duan, Zhaoyin Wang, Mengzhen Xu, and Kang Zhang. Effect of streambed sediment on benthic ecology. *International Journal of Sediment Research*, 24(3): 325–338, September 2009. ISSN 1001-6279. doi: 10.1016/S1001-6279(10)60007-8.

38. E. K. Kupriyanova and C. F. Bailey. The Disturbance Effect of Largemouth Bass Nesting on a Benthic Macroinvertebrate Community. *Journal of Freshwater Ecology*, 13(3):333–341, September 1998. ISSN 0270-5060. doi: 10.1080/02705060.1998.9663626.

39. Emily Y. Campbell, Richard W. Merritt, Kenneth W. Cummins, and M. Eric Benbow. Spatial and Temporal Variability of Macroinvertebrates in Spawning and Non-Spawning Habitats during a Salmon Run in Southeast Alaska. *PLOS ONE*, 7(6): e39254, June 2012. ISSN 1932-6203. doi: 10.1371/journal.pone.0039254.

40. Jacqueline B. Barnes, Ian P. Vaughan, and Steve J. Ormerod. Reappraising the effects of habitat structure on river macroinvertebrates. *Freshwater Biology*, 58(10): 2154–2167, 2013. ISSN 1365-2427. doi: 10.1111/fwb.12198.

41. Y. L. Lau and D. Liu. Effect of flow rate on biofilm accumulation in open channels. *Water Research*, 27(3):355–360, March 1993. ISSN 0043-1354. doi: 10.1016/0043-1354(93)90034-F.

42. R. R. Horner and E. B. Welch. Stream Periphyton Development in Relation to Current Velocity and Nutrients. *Canadian Journal of Fisheries and Aquatic Sciences*, April 2011. doi: 10.1139/f81-062.

43. Michael S. Parker. Effect of Substrate Composition on Detritus Accumulation and Macroinvertebrate Distribution in a Southern Nevada Desert Stream. *The Southwestern Naturalist*, 34(2):181–187, 1989. ISSN 0038-4909. doi: 10.2307/3671727.

44. Ivan Habdija, Biserka Primc Habdija, Renata Matoničkin, Mladen Kučinić, Ines Radanović, Marko Miliša, and Zlatko Mihaljević. Current velocity and food supply as factors affecting the composition of macroinvertebrates in bryophyte habitats in karst running water. *Biologia (Bratislava)*, 59(5):577, 2004.

45. Andrew J. Brooks and Tim Haeusler. Invertebrate responses to flow: Trait-velocity relationships during low and moderate flows. *Hydrobiologia*, 773(1):23–34, June 2016. ISSN 0018-8158, 1573-5117. doi: 10.1007/s10750-016-2676-z.

46. Leah A. Bêche, Eric P. Mcelravy, and Vincent H. Resh. Long-term seasonal variation in the biological traits of benthic-macroinvertebrates in two Mediterranean-climate streams in California, U.S.A. *Freshwater Biology*, 51(1):56–75, 2006. ISSN 1365-2427. doi: 10.1111/j.1365-2427.2005.01473.x.

47. Colin Townsend, Sylvain Dolédec, and Mike Scarsbrook. Species traits in relation to temporal and spatial heterogeneity in streams: A test of habitat templet theory. *Freshwater Biology*, 37(2):367–387, 1997. ISSN 1365-2427. doi: 10.1046/j.1365-2427.1997.00166.x.

48. Simon F. Thrush, Judi E. Hewitt, Vonda J. Cummings, Malcolm O. Green, Greig A. Funnell, and Michelle R. Wilkinson. The Generality of Field Experiments: Interactions Between Local and Broad-Scale Processes. *Ecology*, 81(2):399–415, 2000. ISSN 1939-9170. doi: 10.1890/0012-9658(2000)081[0399:TGOFEI]2.0.CO;2.

49. A. J. Underwood. Beyond BACI: The detection of environmental impacts on populations in the real, but variable, world. *Journal of Experimental Marine Biology and Ecology*, 161(2):145–178, October 1992. ISSN 0022-0981. doi: 10.1016/0022-0981(92)90094-Q.

50. Robert E Bilby, Brian R Fransen, and Peter A Bisson. Incorporation of nitrogen and carbon from spawning coho salmon into the trophic system of small streams: Evidence

from stable isotopes. *Canadian Journal of Fisheries and Aquatic Sciences*, 53(1): 164–173, January 1996. ISSN 0706-652X. doi: 10.1139/f95-159.

51. David J. Janetski, Dominic T. Chaloner, Scott D. Tiegs, and Gary A. Lamberti. Pacific salmon effects on stream ecosystems: A quantitative synthesis. *Oecologia*, 159(3): 583–595, March 2009. ISSN 1432-1939. doi: 10.1007/s00442-008-1249-x.

52. Jan J. Verspoor, Douglas C. Braun, Morgan M. Stubbs, and John D. Reynolds. Persistent ecological effects of a salmon-derived nutrient pulse on stream invertebrate communities. *Ecosphere*, 2(2):art18, 2011. ISSN 2150-8925. doi: 10.1890/ES10-00011.1.

53. Bernard R. Smith. Sea lampreys in the Great Lakes of North America. pages 207–247, 1971.

54. Vernon C. Applegate. Natural history of the sea lamprey, *Petromyzon marinus*, in Michigan. Federal Government Series 55, U.S. Fish and Wildlife Service, Ann Arbor, Michigan: University of Michigan Library, 1950.

55. Daniel Weaver, Stephen M. Coghlan, and Joseph D. Zydlewski. Sea Lamprey Carcasses Exert Local and Variable Food Web Effects in a Nutrient-limited Atlantic Coastal Stream. *Canadian Journal of Fisheries and Aquatic Sciences*, 73, May 2016. doi: 10.1139/cjfas-2015-0506.

56. J. Bruce Wallace and Jackson R. Webster. The Role of Macroinvertebrates in Stream Ecosystem Function. *Annual Review of Entomology*, 41(1):115–139, 1996. doi: 10.1146/annurev.en.41.010196.000555.

57. J. Bruce Wallace and Martin E. Gurtz. Response of Baetis Mayflies (Ephemeroptera) to Catchment Logging. *The American Midland Naturalist*, 115(1):25–41, 1986. ISSN 0003-0031. doi: 10.2307/2425834.

58. Nancy B. Grimm. Role of Macroinvertebrates in Nitrogen Dynamics of a Desert Stream. *Ecology*, 69(6):1884–1893, 1988. ISSN 1939-9170. doi: 10.2307/1941165.

59. Mark D. Hunter, Sina Adl, Catherine M. Pringle, and David C. Coleman. Relative effects of macroinvertebrates and habitat on the chemistry of litter during decomposition. *Pedobiologia*, 47(2):101–115, January 2003. ISSN 0031-4056. doi: 10.1078/0031-4056-00174.

60. Walter R. Hill, Harry L. Boston, and Alan D. Steinman. Grazers and Nutrients Simultaneously Limit Lotic Primary Productivity. *Canadian Journal of Fisheries and Aquatic Sciences*, 49(3):504–512, March 1992. ISSN 0706-652X. doi: 10.1139/f92-059.

61. Verónica Ferreira, Eric Chauvet, and Cristina Canhoto. Effects of experimental warming, litter species, and presence of macroinvertebrates on litter decomposition and associated decomposers in a temperate mountain stream. *Canadian Journal of Fisheries and Aquatic Sciences*, 72(2):206–216, February 2015. ISSN 0706-652X. doi: 10.1139/cjfas-2014-0119.

62. Thendo Mutshekwa, Ross N. Cuthbert, Ryan J. Wasserman, Florence M. Murungweni, and Tatenda Dalu. Macroinvertebrate colonisation associated with native and invasive leaf litter decomposition. *Knowledge & Management of Aquatic Ecosystems*, (421):32, 2020. ISSN 1961-9502. doi: 10.1051/kmae/2020025.

63. N H Anderson and J R Sedell. Detritus Processing by Macroinvertebrates in Stream Ecosystems. *Annual Review of Entomology*, 24(1):351–377, 1979. doi: 10.1146/annurev.en.24.010179.002031.

64. Fredric M. Windsor, Rosie M. Tilley, Charles R. Tyler, and Steve J. Ormerod. Microplastic ingestion by riverine macroinvertebrates. *Science of The Total*

Environment, 646:68–74, January 2019. ISSN 0048-9697. doi: 10.1016/j.scitotenv.2018.07.271.

65. Florian Mermillod-Blondin, Jean-Paul Gaudet, Magali Gérino, Gaston Desrosiers, and Michel Creuzé des Châtelliers. Influence of macroinvertebrates on physico-chemical and microbial processes in hyporheic sediments. *Hydrological Processes*, 17(4): 779–794, 2003. ISSN 1099-1085. doi: 10.1002/hyp.1165.

66. Kenneth W. Cummins. Hynes, H. B. N. The ecology of running waters. *Limnology and Oceanography*, 16(3):593–594, 1971. ISSN 1939-5590. doi: 10.4319/lo.1971.16.3.0593.

67. Manuela Abelho and M. A. S. Graça. Litter in a first–order stream of a temperate deciduous forest (Margaraça Forest, central Portugal). *Hydrobiologia*, 386(1):147–152, November 1998. ISSN 1573-5117. doi: 10.1023/A:1003532921432.

68. Michael Monaghan, Steven Thomas, G Minshall, Denis Newbold, and Colbert Cushing. The influence of filter-feeding benthic macroinvertebrates on the transport and deposition of particulate organic matter and diatoms in two streams. *Limnology and oceanography*, 46:1091–1099, July 2001. doi: 10.4319/lo.2001.46.5.1091.

69. Andrew F. Brown and Mark Diamond. The consumption of rainbow trout (*Salmo gairdneri* Richardson) eggs by macroinvertebrates in the field. *Freshwater Biology*, 14 (2):211–215, 1984. ISSN 1365-2427. doi: 10.1111/j.1365-2427.1984.tb00036.x.

70. Chris A. Mills. Egg population dynamics of naturally spawning dace, *Leuciscus leuciscus* (L.). *Environmental Biology of Fishes*, 6(2):151–158, May 1981. ISSN 1573-5133. doi: 10.1007/BF00002778.

71. ECP. Ecology and Fish Population Biology Facility. *INRAE*, 2018. doi: 10.15454/1.5572402068944548E12.

Supporting information

Fig 10. Median of the abundances for the 10 most abundant taxa within each zone (Control, Pit, Mound).

Fig 11. Number of exclusive taxa in mound or pit compared to the control.
With $^{**}P \leq 0.01$.

Figure

Click here to access/download;Figure;Fig1.tiff

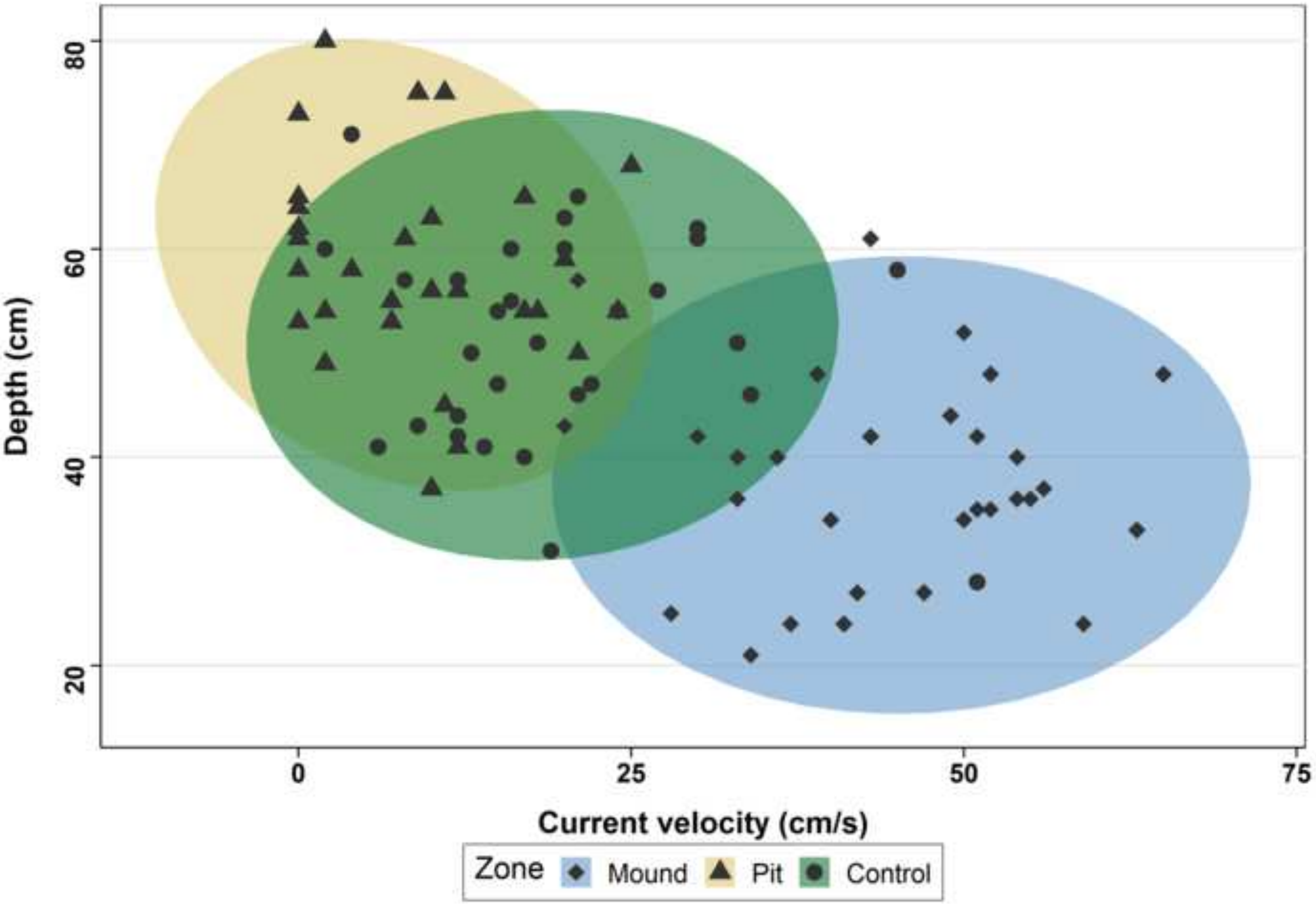

Figure

Click here to access/download;Figure;Fig2.tiff

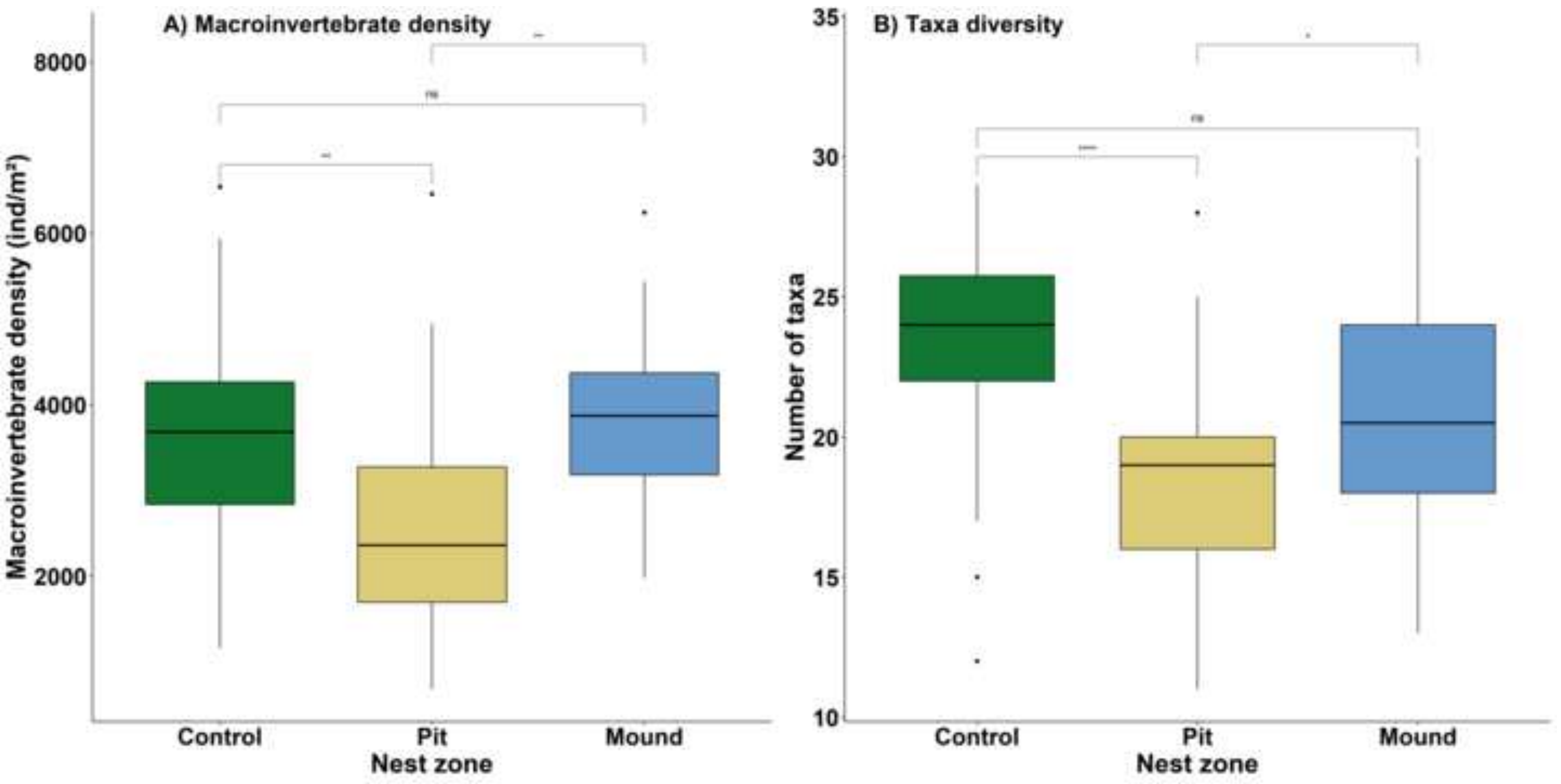

 Click here to access/download;Figure;Fig3.tiff

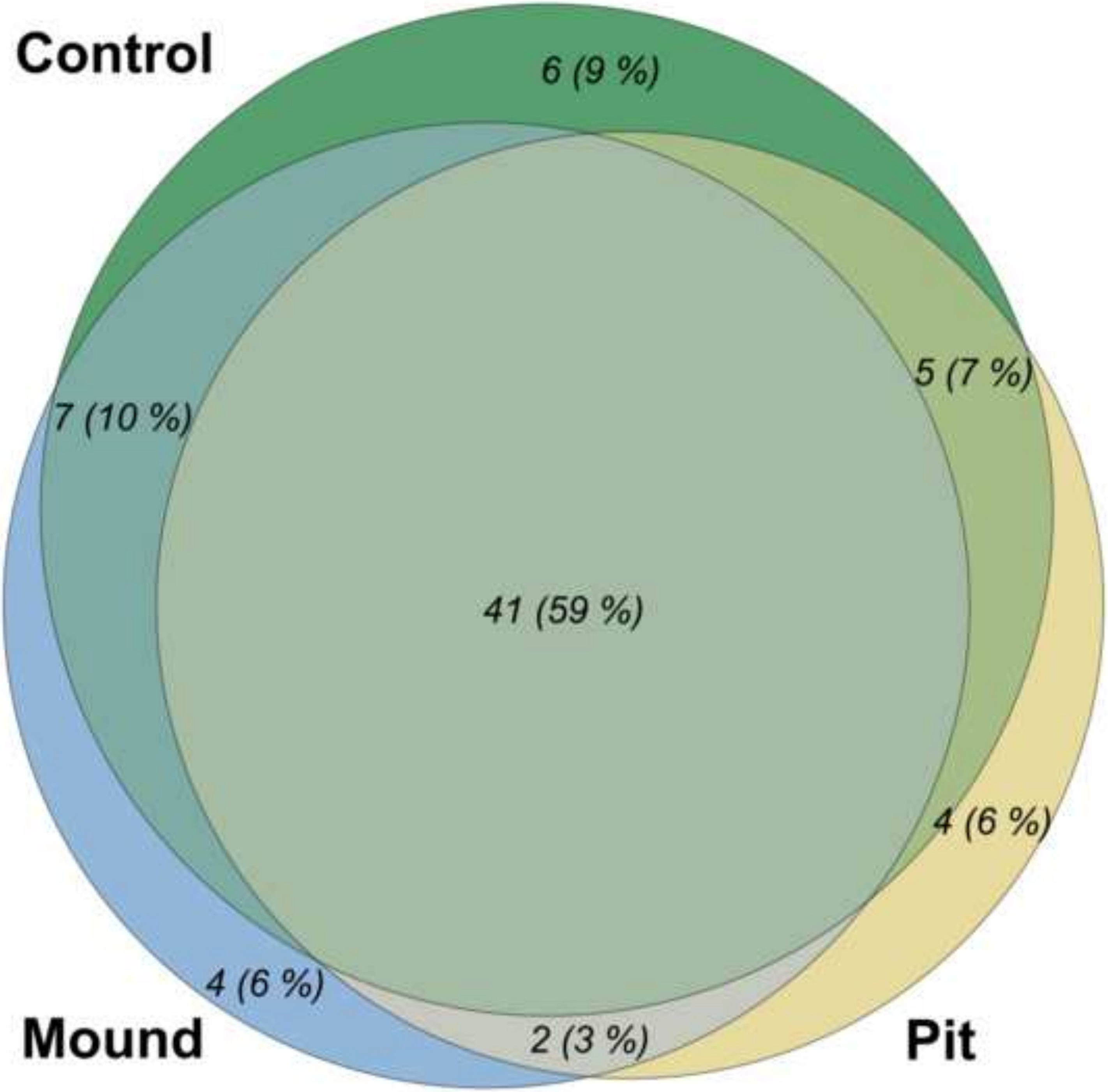

 Click here to access/download;Figure;Fig4.tiff

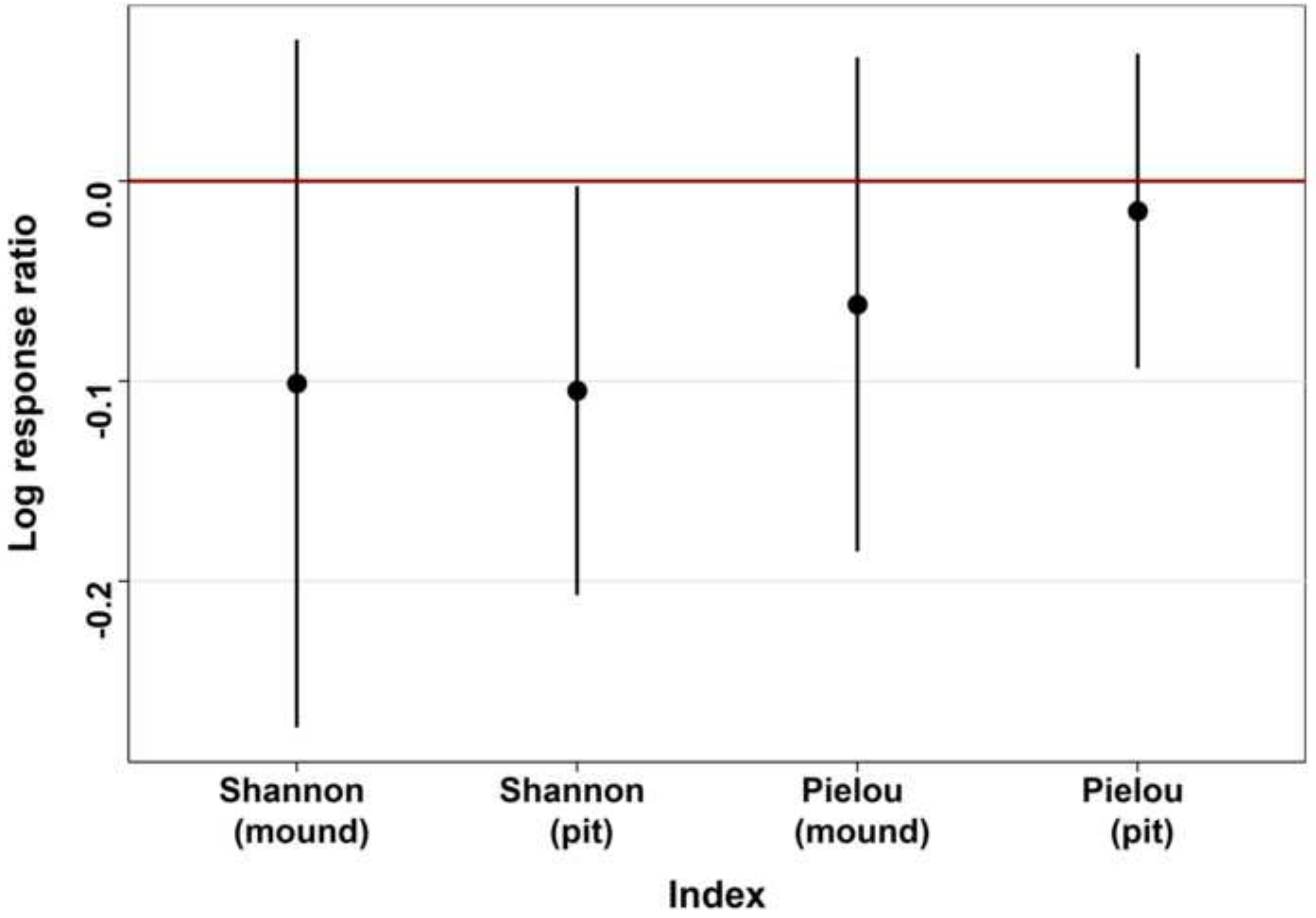

Figure Click here to access/download;Figure;Fig5.tiff

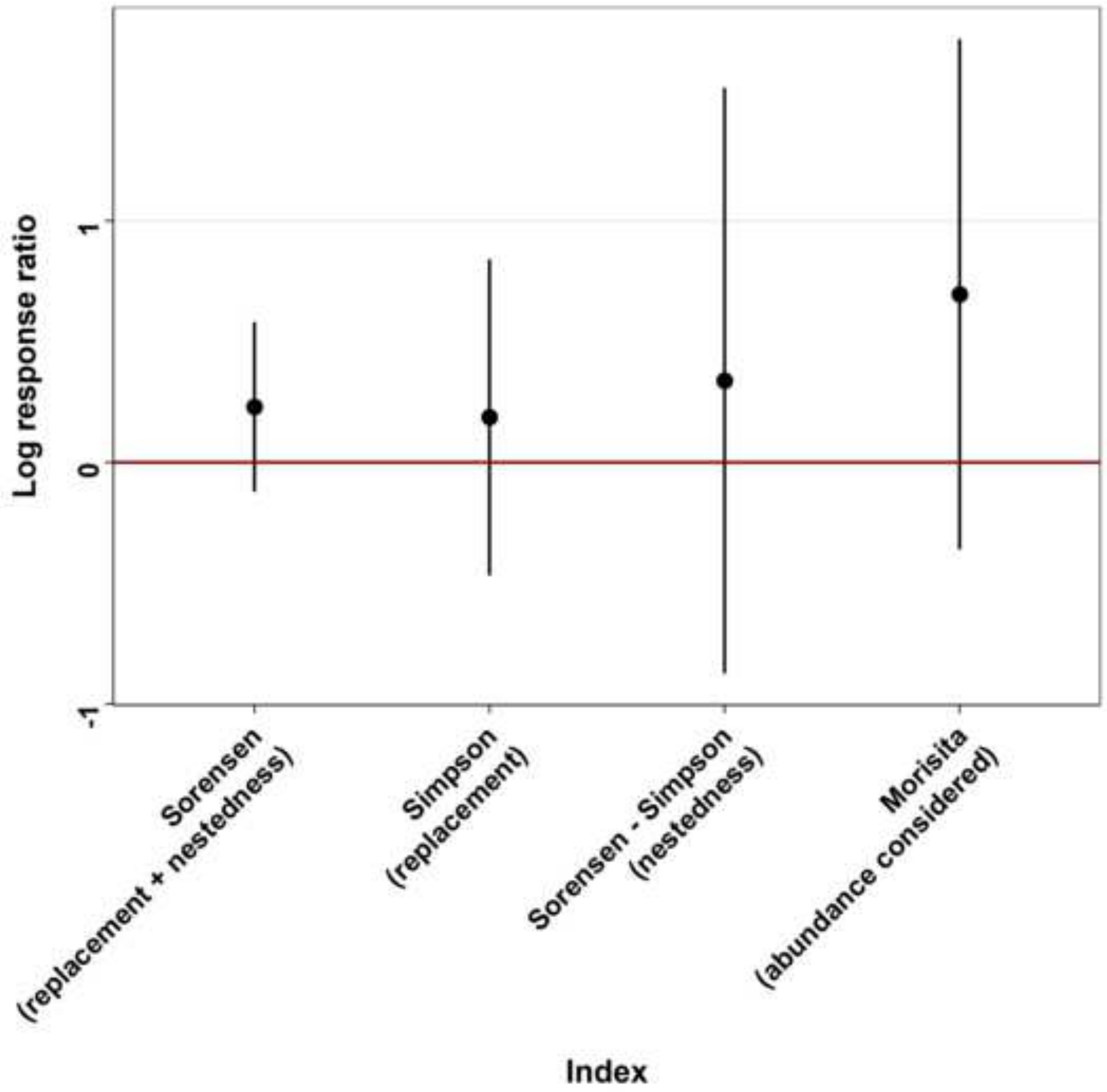

Figure

Click here to access/download;Figure;Fig6.tiff

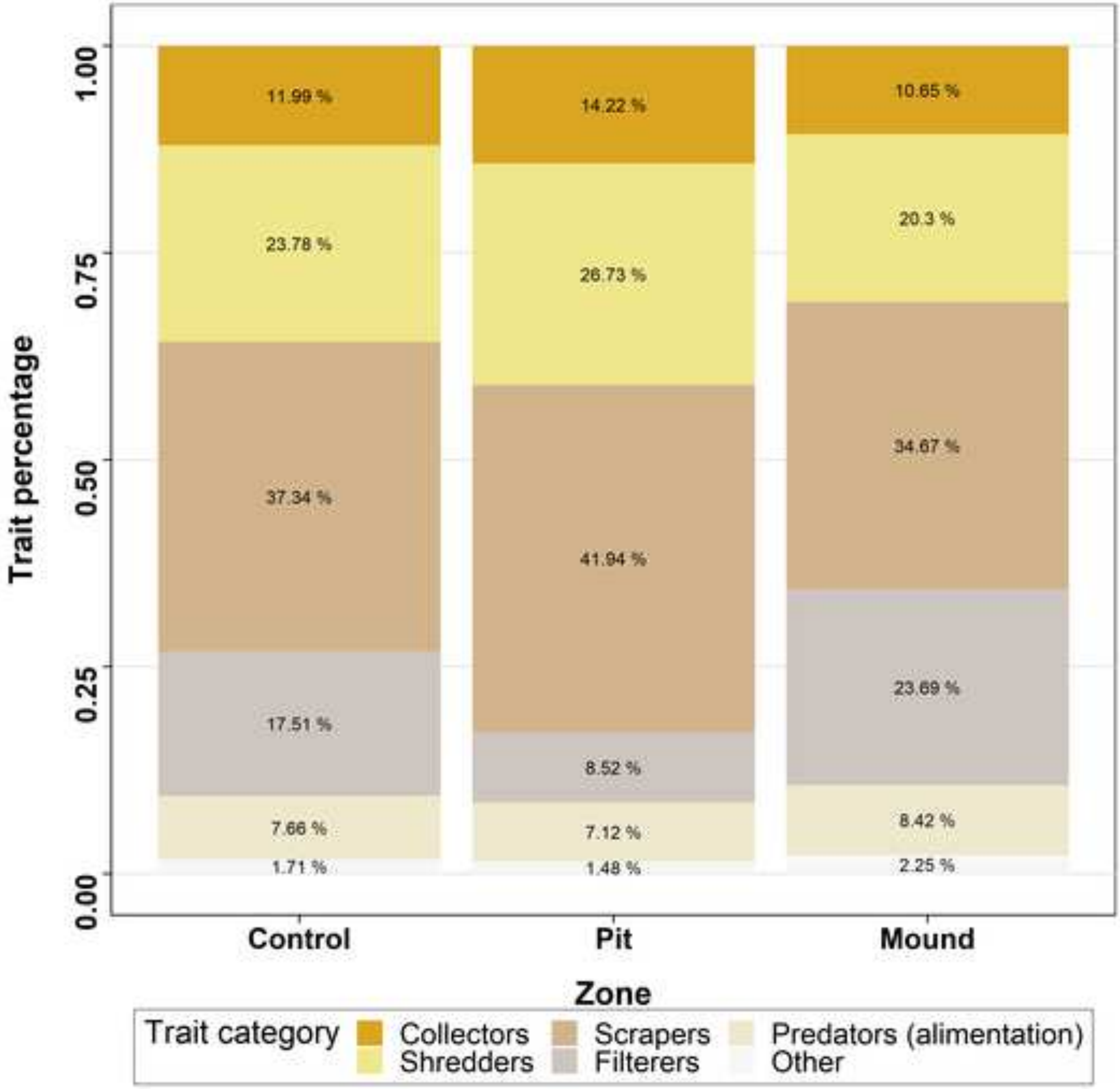

Figure Click here to access/download;Figure;Fig7.tiff

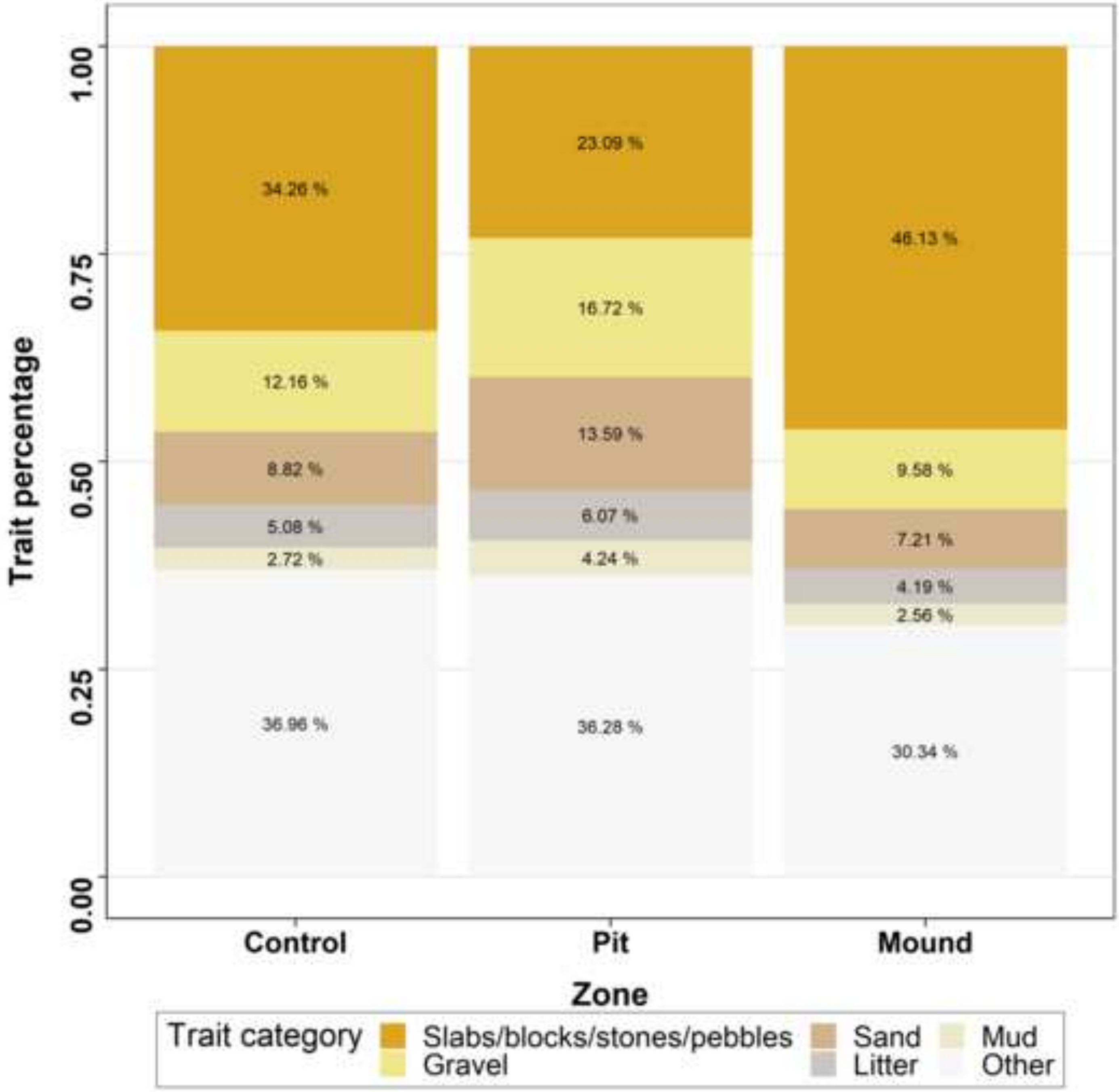

 Click here to access/download;Figure;Fig8.tiff

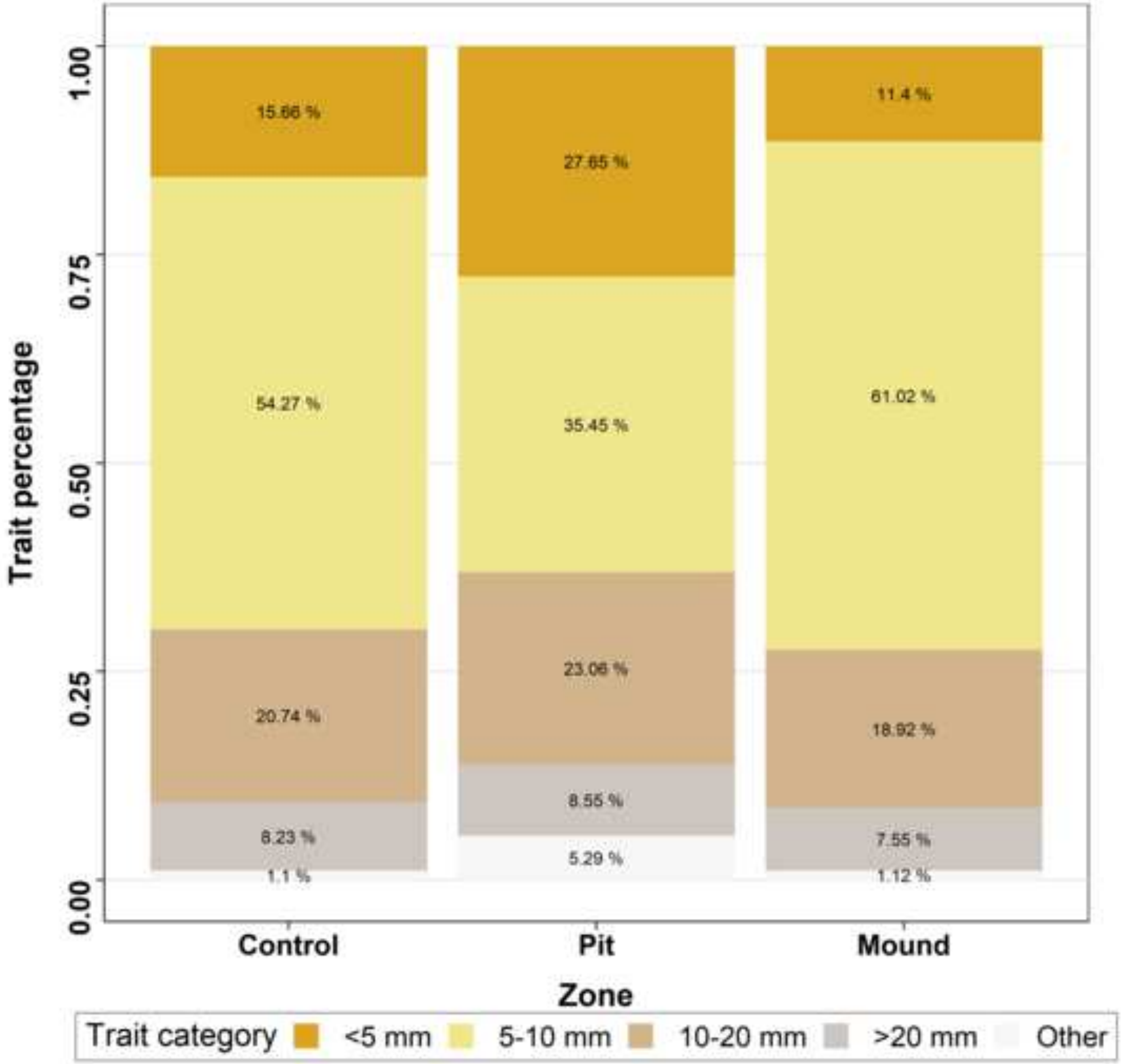

Figure

Click here to access/download;Figure;Fig9.tiff

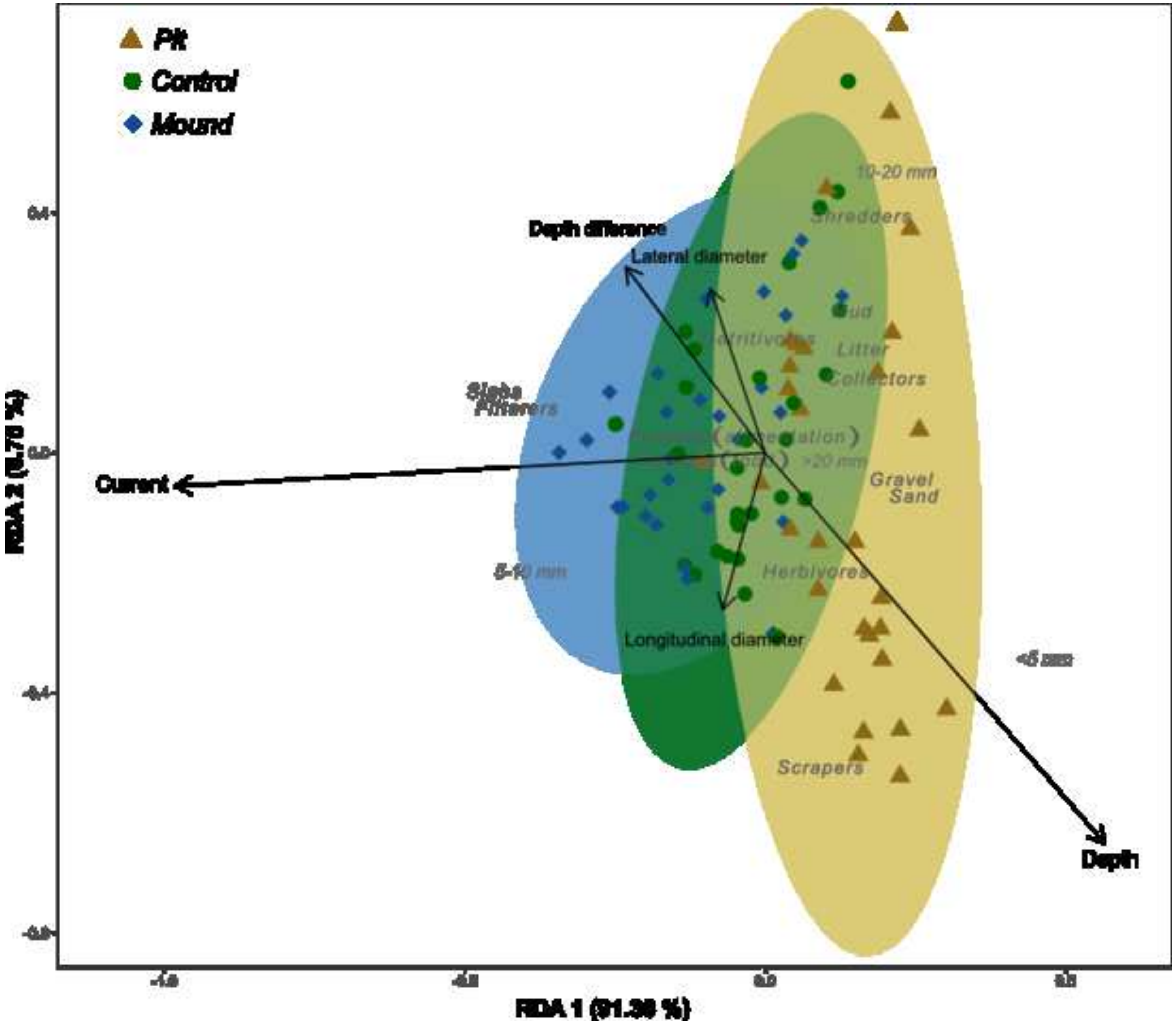

Click here to access/download
Supporting Information
Fig10.tiff

Click here to access/download
Supporting Information
Fig11.tiff
